# Elucidating the genomic architecture of Asian *EGFR*-mutant lung adenocarcinoma through multi-region exome sequencing

**DOI:** 10.1038/s41467-017-02584-z

**Published:** 2018-01-15

**Authors:** Rahul Nahar, Weiwei Zhai, Tong Zhang, Angela Takano, Alexis J. Khng, Yin Yeng Lee, Xingliang Liu, Chong Hee Lim, Tina P. T. Koh, Zaw Win Aung, Tony Kiat Hon Lim, Lavanya Veeravalli, Ju Yuan, Audrey S. M. Teo, Cheryl X. Chan, Huay Mei Poh, Ivan M. L. Chua, Audrey Ann Liew, Dawn Ping Xi Lau, Xue Lin Kwang, Chee Keong Toh, Wan-Teck Lim, Bing Lim, Wai Leong Tam, Eng-Huat Tan, Axel M. Hillmer, Daniel S. W. Tan

**Affiliations:** 1 Cancer Therapeutics and Stratified Oncology, Genome Institute of Singapore, Singapore, 138672 Singapore; 2 Human Genetics, Genome Institute of Singapore, Singapore, 138672 Singapore; 3 School of Biological Sciences, Nanyang Technological University, Singapore, 637551 Singapore; 4 Department of Pathology, Singapore General Hospital, Singapore, 169608 Singapore; 5 Department of Cardiothoracic Surgery, National Heart Centre Singapore, Singapore, 169609 Singapore; 6 Division of Clinical Trials and Epidemiological Sciences, National Cancer Centre Singapore, Singapore, 169610 Singapore; 7 Research Pipeline Development, Genome Institute of Singapore, Singapore, 138672 Singapore; 8 Cancer Stem Cell Biology, Genome Institute of Singapore, Singapore, 138672 Singapore; 9 Next Generation Sequencing Platform, Genome Institute of Singapore, Singapore, 138672 Singapore; 10 Division of Medical Oncology, National Cancer Centre Singapore, Singapore, 169610 Singapore; 11 Cancer Therapeutics Research Laboratory, Division of Medical Sciences, National Cancer Centre Singapore, Singapore, 169610 Singapore; 12 Institute of Pathology, University Hospital Cologne, 50937 Cologne, Germany

## Abstract

*EGFR*-mutant lung adenocarcinomas (LUAD) display diverse clinical trajectories and are characterized by rapid but short-lived responses to EGFR tyrosine kinase inhibitors (TKIs). Through sequencing of 79 spatially distinct regions from 16 early stage tumors, we show that despite low mutation burdens, *EGFR*-mutant Asian LUADs unexpectedly exhibit a complex genomic landscape with frequent and early whole-genome doubling, aneuploidy, and high clonal diversity. Multiple truncal alterations, including *TP53* mutations and loss of *CDKN2A* and *RB1*, converge on cell cycle dysregulation, with late sector-specific high-amplitude amplifications and deletions that potentially beget drug resistant clones. We highlight the association between genomic architecture and clinical phenotypes, such as co-occurring truncal drivers and primary TKI resistance. Through comparative analysis with published smoking-related LUAD, we postulate that the high intra-tumor heterogeneity observed in Asian *EGFR*-mutant LUAD may be contributed by an early dominant driver, genomic instability, and low background mutation rates.

## Abstract (web summary)

EGFR mutant lung adenocarcinoma (LUAD) exhibit diverse clinical outcomes in response to targeted therapies. Here the authors show that these LUADs involve a complex genomic landscape with high intratumor heterogeneity, providing insights into the evolutionary trajectory of oncogene-driven LUAD and potential mediators of EGFR TKI resistance.

## Introduction

Although comprehensive genomic sequencing studies have identified recurrent somatic alterations in LUAD^1–8^, majority has been based on single-tissue samples and lack the resolution to evaluate clonal architecture. Further, certain clinical phenotypes, such as never-smoker Asian *EGFR*-mutant LUAD are under-represented in these cohorts^1–8^. Activating mutations in the epidermal growth factor receptor (*EGFR*) are the most common therapeutically tractable driver mutation in lung adenocarcinomas (LUAD) with distinct ethnic differences, occurring at higher frequencies in Asians (40–60%) compared to Caucasians (7–10%)^9–12^. Due to the preponderance of never smokers, *EGFR*-mutant LUADs are often associated with low-mutation burdens. On the contrary, the copy number landscape has been shown to harbor considerable genomic complexity^13–15^, although the extent to which these observations are confounded by intra-tumor heterogeneity (ITH) is unclear.

While it is feasible to computationally resolve clonal composition in single-tissue samples^16–18^, these methods lack sensitivity and specificity especially in tumors with high ITH, where regionally dominant clones may exist. More recently, multi-region sequencing studies on smoker dominated Caucasian non-small cell lung cancers (NSCLC) have revealed high-mutation burden and low ITH^19–21^—primarily attributed to inordinately long trunks (representing mutations shared by all regions of a tumor) as a result of accruing multiple passenger and driver alterations from chronic tobacco exposure. On the other hand, late diversification was contributed by branch/private driver alterations (mutations present in few but not all regions of tumor/present in single region of tumor) and increased APOBEC activity, representing evolutionary processes that are potentially amenable to therapeutic targeting^16,19^.

The extent of ITH, and the factors that drive cancer evolution, is of clinical interest as it has been inextricably linked to treatment failure^22,23^. In *EGFR*-mutant LUAD, selection pressures imposed by targeted therapies can either result in expansion of pre-existing rare TKI resistant subclones (e.g., cells harboring EGFR T790M or *MET* amplifications) or be acquired stochastically^24–26^. The rapid emergence of resistance to EGFR TKIs^27,28^ seems counter intuitive in the context of low-mutation burden, and the impact of intra-tumor heterogeneity on the extent of tumor shrinkage and eventual emergence of drug resistance is not well established.

Here we present the first comprehensive description of the genomic architecture of *EGFR*-mutant LUAD through multi-region exome sequencing and SNP arrays on 16 tumors. Despite low-mutation burdens in these predominantly never-smoker oncogene-driven LUADs, we demonstrate an under-appreciated level of genomic complexity, both in terms of copy number landscape, as well as relatively high-proportional ITH due to early diversification in these tumors. Through integrative genomics, we show that early *EGFR* and *TP53* mutations are often followed by genome doubling events, with ongoing genomic instability typified by a variegated copy number landscape and late high-amplitude amplifications and deletions. We further highlight how multiple co-occurring drivers may portend poor clinical outcomes, including primary EGFR TKI resistance. By comparing the clonal architecture of our *EGFR*-mutant LUAD with that of previously published smoking-related LUAD, we provide insights into determinants of ITH and suggest that the evolutionary trajectories of LUADs are shaped by cumulative effects of background mutation rates, strength and timing of driver mutations and ongoing genomic instability.

## Results

### Spatio-temporal relationship of mutations relative to *EGFR*

**Fig. 1:**
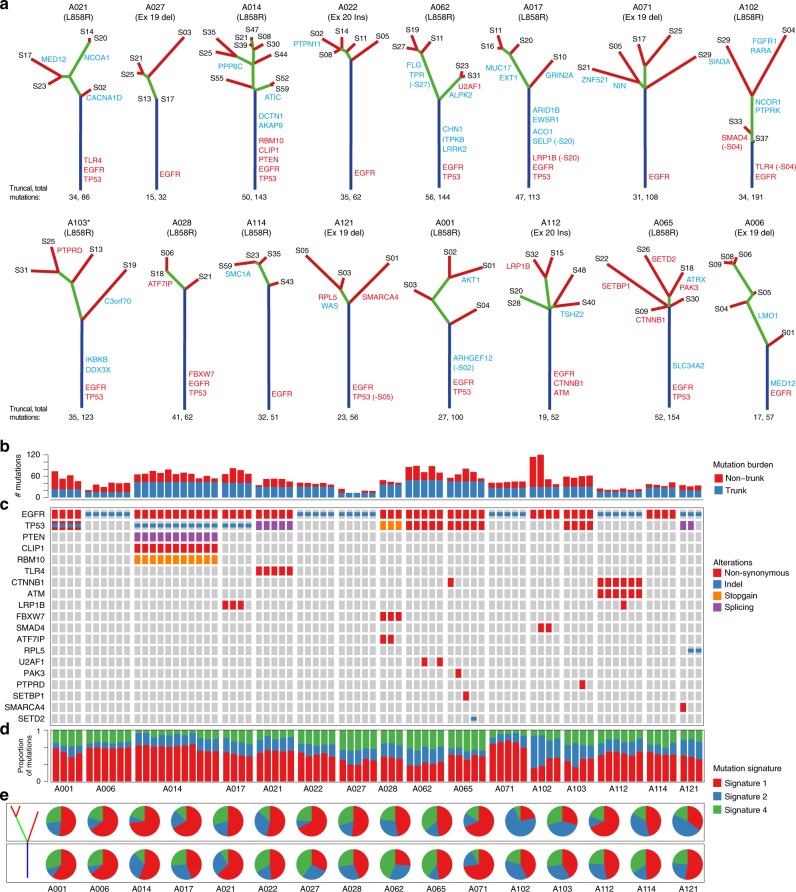
Landscape of clonal and subclonal mutations in Asian *EGFR*-mutant tumors. **a** Phylogenetic trees generated for the 16 Asian *EGFR*-mutant LUADs. Trunks, branches and tips are depicted in blue, green, and red, respectively, while non-silent mutations carrying LUAD specific drivers are in red and other cancer drivers are in blue (Methods section). All patients are never-smokers except A103 (marked *) who was a light ex-smoker. Truncal mutation burden followed by total mutation burden for all sectors is indicated below the trees. **b** Bar plot representing truncal and non-truncal mutation burden per sector. **c** Oncoprint heatmap for mutations in LUAD drivers depicting the presence (see color legend) or absence (gray box) and type of non-silent mutation. **d** Proportions of the three mutation signatures identified for each sector. Signature numbers are according to the COSMIC nomenclature. **e** Pie charts representing contribution of the three mutation signatures in early (trunk) and late (branch/private) mutations

We subjected 16 surgically resected stage I–II treatment naive *EGFR*-mutation positive Asian LUAD cases (15 never smokers) to multi-sector whole-exome sequencing. A total of 79 sectors with 3–11 regions from each tumor were sequenced (mean depth 114X; Supplementary Fig. 1; Supplementary Table 1). Somatic single-nucleotide variants (SNVs) and indels were identified and subjected to target deep sequencing (mean depth of 3860X). In all, 1450 SNVs and 71 indels were confirmed, affecting exons of 1318 genes (Supplementary Data 1), from which phylogenetic trees and mutation heatmaps were generated (Fig. 1a, b; Supplementary Fig. 2).

*EGFR* mutations were confirmed to be truncal events (mutations present in all sectors of a tumor) in every case regardless of the mutation type (L858R, exon 19 deletion or exon 20 insertion), underscoring its role as an early tumor initiating driver event (Fig. 1a, Supplementary Data 2). Besides *EGFR*, *TP53* was the most recurrently mutated gene with mutations in 9 out of 16 tumors, of which eight were truncal events (Fig. 1a, c; Supplementary Data 1, 2). Only 17 other LUAD-specific driver genes (those found to be recurrently mutated in LUAD; Methods section) were found mutated, of which 7 (<50%) featured as truncal events in 4/16 tumors (Fig. 1c). Further, just two of these 17 driver genes (*LRP1B* and *CTNNB1*) were recurrently mutated across two patients (Fig. 1a, c, Supplementary Data 1, 2). Interestingly, both *CTNNB1* mutations (S37C and K335I, Supplementary Data 1) are known to be oncogenic and deregulate beta-catenin activity^29,30^ possibly contributing to *EGFR*-mediated tumorigenesis^31^. In addition, we find infrequent mutations that have recently been shown to have functional roles. For example, the private mutation affecting D323 residue of AKT1 (found in A001, Fig. 1a, Supplementary Data 2) has been shown to be activating/oncogenic^32^ contributing to erlotinib resistance in *EGFR*-mutant PC-9 cells^33^. Loss-of-function of MED12 has been implicated in resistance to EGFR TKIs^34^ suggesting that the truncal *MED12* frameshift deletions in A006 and the private *MED12* mis-sense mutation (predicted to be damaging; Supplementary Data 1, 2) in A021 might be candidates for resistance inducing mutations. However, we did not find any common resistance mutations like the T790M in *EGFR*, in any sector, likely due to their rare occurrence in treatment naive samples that are beyond the detection limits of our sequencing parameters^24^.

### Multi-region sequencing reveals high ITH in *EGFR*-mutant LUAD

Using the percentage of branch/private mutations—a common measurement of ITH^19,20,35^ (we term this proportional ITH or pITH), we found a median of 62.3% heterogeneity (range: 32.26–82.2%) in our largely never-smoker Asian *EGFR*-mutant LUADs, contrary to previous findings of low ITH in LUAD (~30% branch mutations; Fig. 1a, b, Supplementary Fig. 2, Supplementary Data 2)^19,20^. To eliminate any biases arising from different analysis pipelines, we re-analysed the data from the two earlier reports using our pipelines (Supplementary Fig. 3, Supplementary Table 2) and confirmed the differences in pITH even after controlling for the number of sectors per tumor (Supplementary Fig. 4). Consistent with the higher pITH, we found an average increase of 37% in mutation burden upon sequencing three random sectors in *EGFR* mutation positive Asian LUAD compared to only 17% increase in smoker dominated Caucasian LUAD (Supplementary Fig. 5), underscoring the higher relative burden of branch/private mutations in the former.

### APOBEC activity is infrequently observed in *EGFR*-mutant LUAD

We next examined the mutational signatures associated with early and late genetic events in Asian *EGFR-*mutant LUAD. Among the three mutation signatures identified in our patients (Supplementary Fig. 6) the age-associated molecular clock like signature-1^36,37^ dominated the mutational landscape of these 16 *EGFR*-mutant patients including the light ex-smoker A103 (Fig. 1d). The distribution of signatures between early (trunk) and late events (branch/private) was heterogeneous across patients (Fig. 1e) and collectively we did not find significant change for any signature between the early and late events (signature-1: *P* = 0.43; signature-2: *P* = 0.62; signature-4: *P* = 0.08, paired *t*-test). However, 4 of 16 patients (A102, A103, A114, and A121) demonstrated relatively higher contribution of APOBEC activity (signature-2) in late subclonal events (Fig. 1e). Of note, marked spatial heterogeneity was observed in one patient (A102)—a *TP53* wild-type non-smoker, where two out of four sectors showed considerably increased contribution of APOBEC signature and disproportionately higher sector-specific mutational burden (Fig. 1b, d, e). These observations support the role of increased activity of APOBEC family of enzymes as a putative mechanism driving subclonal diversification^16,19,20^, although only in a minority of *EGFR*-mutant cases.

### *EGFR*-mutant LUAD display a variegated copy number landscape

**Fig. 2:**
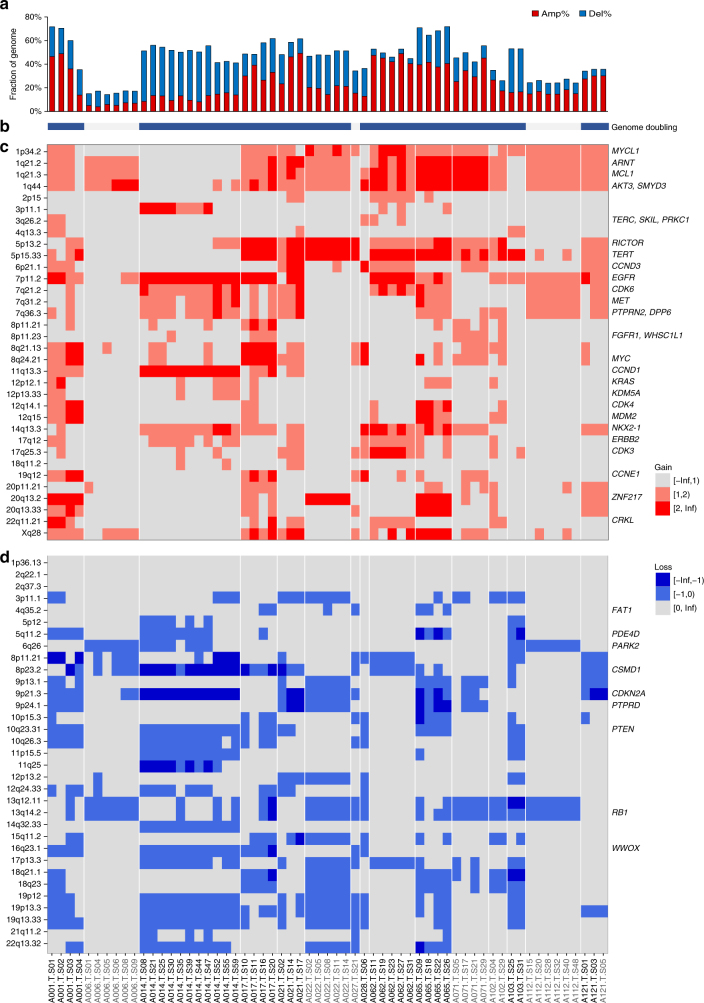
Genomic instability and variegated copy number landscape of *EGFR*-mutant tumors. **a** Bar plot representing the fraction of genome altered by copy number alterations relative to ploidy of the sector, which is termed as the genomic instability index (GII). **b** Bar showing genome doubling status. Blue indicates significant evidence for genome doubling (Supplementary Data 3, Methods section) and gray indicates no genome doubling. **c** Heatmap depicting gains in known driver or recurrently amplified cytobands in LUAD (Methods section). Light red represents gain in one copy beyond the ploidy while dark red represents gain in ≥2 copies beyond the ploidy. **d** Heatmap depicting losses in known driver or recurrently amplified cytobands in LUAD (Methods section). Light blue represents loss of one copy relative to the ploidy while dark blue represents loss of ≥2 copies beyond the ploidy. Samples for all panels are as depicted in **d**. *TP53* wild-type samples are depicted in gray

We successfully profiled somatic copy number alterations (SCNA) for 61 of the 79 tumor sectors across 15 patients (Fig. 2, Supplementary Fig. 7). Using a genome instability index (GII, defined as fraction of the genome altered by SCNAs, copy change ≥1 relative to ploidy; see Methods section), we found that majority of tumors showed moderate to high-genomic instability (median of 48.1% per tumor sector, Fig. 2a), as well as frequent whole-genome doubling (WGD) events (12 of 15 tumors, except A006, A027, and A112; Fig. 2b, Supplementary Data 3). Interestingly, we find WGD to be a truncal event (Fig. 2b, Supplementary Data 3) wherever present, suggesting them to occur early in tumorigenesis, consistent with its implications as a common route leading to genomic and chromosomal instability (CIN), fueling intra-tumor heterogeneity^6,38,39^. Using the eleven tumors with SCNA data in at least three sectors, we observed 40.5% of cytobands and 41.35% of genes to be affected by late branch or private copy number alterations (Supplementary Fig. 8; Supplementary Data 4, 5; Methods section). While we saw little variance in GII scores across sectors (Fig. 2a), majority of the SCNAs contributing were low-copy gains and losses (copy change = 1 relative to ploidy; Supplementary Figs 7, 9). In contrast, while a median of only 7.2% of genome was affected by high-copy gains and losses (copy change ≥2 relative to ploidy; defined as adGII or amplification and deletion based genomic instability index; Supplementary Fig. 9), we observed a significantly higher variance in adGII scores across sectors of a tumor compared to GII scores (*P* = 5 × 10^−4^, Welch’s *t*-test; Supplementary Fig. 10) suggesting continuous evolution of copy number landscape with late increase in amplitude of the alterations. Focusing on genes where amplifications have been reported as putative resistance mechanisms to EGFR TKIs^25,40^, we find low-copy gains for *MET*, *ERBB2*, and *HGF* genes in 12/15 patients (Supplementary Fig. 11). While some were truncal events, five patients displayed ≥5 copies in either of these three genes in at least one sector confirming that these amplifications do pre-exist subclonally in some tumors (Supplementary Fig. 11, Supplementary Data 4), and may contribute to a drug tolerant state.

We next estimated the timing of recurrent truncal mutations relative to WGD and copy number alterations using a published algorithm^16^. The inferred cancer cell fractions and mutant allele copy numbers suggest that *EGFR* and *TP53* mutations occurred prior to WGD and local SCNA (Supplementary Data 6), underscoring the founding role of these two drivers during tumorigenesis. Notably, in the nine tumors harboring mutations in *TP53*, all had undergone WGD (compared to 3 out of 6 in *TP53* wild type (wt)) (Fig. 2b, Supplementary Data 3) and were further associated with significantly higher genomic instability (*P* = 0.0131, Welch’s *t*-test; Supplementary Fig. 12, Fig. 2a). Overall, our data reveal how *EGFR*-mutant LUAD can harbor a complex copy number landscape that can be influenced by *TP53* mutation status—and undergoes continuous evolution over time with early low-copy gains and losses followed by late high-amplitude changes.

### Early SCNA converge on disruption of cell cycle control

We next examined the recurrent copy number changes, focusing on the cytobands with recurrent SCNAs and driver cytobands identified in previous large scale studies^4,6–8,41^. We found gains in multiple cytobands from chromosome 1, 5p, and 7p containing important driver genes like *TERT*, *EGFR*, anti-apoptotic *MCL1* and *TP53* inactivator *MDM4* as the most recurring truncal events in more than half of the tumors evaluated for SCNA ITH (Fig. 2c; Supplementary Fig. 7; Supplementary Data 4, 5). Truncal deletion events were observed in regions, such as 13q14.2, 9p21.3 and 10q23.31 containing known tumor suppressors like *RB1*, *CDKN2A*, and *PTEN* (Fig. 2d). Among these, 9p21.3 containing *CDKN2A/2B* carried truncal losses relative to ploidy across six tumors and was the only known driver region with truncal homozygous deletions in two tumors (Fig. 2d; Supplementary Fig. 13a; Supplementary Data 4, 5). Interestingly, all *TP53* wild-type tumors had truncal losses affecting the 13q14.2 region containing *RB1* (Fig. 2d) and four of these tumors had loss of heterozygosity (LOH) containing just single copy of this gene (Supplementary Fig. 13b). In addition to recurrent somatic mutations in *TP53*, we further observed recurrent truncal LOH in 7/11 tumors including those without any mutations (Supplementary Fig. 13c). In *EGFR*-mutant tumors with *TP53* mutations, 6/9 tumors were found to have LOH and loss in copy number in *RB1* region (3 tumors with potentially truncal LOH; Supplementary Fig. 13b). Overall these findings implicate disruption of the *RB1/CDKN2A/TP53* control axis of the cell cycle G1/S and apoptosis checkpoints as an early tumor initiating event in *EGFR*-mutant LUADs.

### Determinants of high ITH in *EGFR* mutant LUAD

**Fig. 3:**
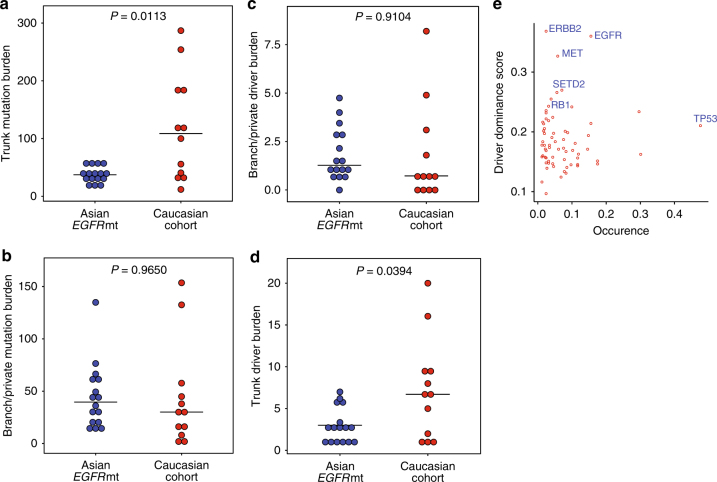
*EGFR* is a dominant driver with few co-drivers. Dot plots comparing mutation burden on **a** trunk and **b** branches between the Asian *EGFR*-mutant and smoker dominated Caucasian cohorts^19, 20^. **c** Dot plot comparing number of branch/private mutated drivers (extended driver list) between the EGFR-mutant and smoker dominated Caucasian cohorts^19, 20^. Welch’s *t*-test was used to compare the two groups. **d** Dot plot showing that *EGFR*-mutant LUADs have significantly fewer truncal drivers (extended driver list; Welch’s *t*-test) compared to smoker Caucasian LUADs. Three random sectors were picked 20 times iteratively and averages of the iterations are represented as circles in **a**–**d**. Horizontal line indicates the median for that cohort. **e** “Driver dominance score” measuring driver self-sufficiency for each of 78 LUAD driver genes calculated across published 412 tumors^3, 4^ is plotted against the fraction of patients carrying the mutated driver

Although lower pITH in the smoker enriched Caucasian cohort (Supplementary Fig. 4) can be explained by the higher number of smoking induced truncal mutations during the life history of a tumor (Fig. 3a), the comparable absolute mutation burden on the branches between smoker Caucasian and our non-smoker cohorts (Fig. 3b) was unexpected. Given that smoking related tumors can harbor up to 10 times the mutation burden of non-smoking counterparts^2,3^, the comparable level of genetic diversity (number of branch mutations) attained by *EGFR*-mutant LUAD was quite striking. In exploring potential reasons for the unexpectedly high-branch mutations in *EGFR*-mutant LUAD, we did not find enrichment for subclonal drivers (Fig. 3c) nor consistent increase in APOBEC-associated mutagenesis, the latter found in only a subset of tumors (Fig. 1d, e).

Based on the fewer truncal drivers observed in *EGFR*-mutant LUAD compared to smoker dominated Caucasian LUAD (Fig. 3d), we surmised that *EGFR* mutations may require fewer co-drivers for clonal expansion. To test whether *EGFR* tends to be dominant (i.e., ‘self-sufficient’) and has less co-drivers across tumors compared to other LUAD drivers, we developed a “driver dominance score”, which measures the number of co-occurring drivers for each defined driver gene per tumor. Drivers with higher dominance score will tend to have less co-drivers across cases (Methods section). Applying this procedure across 412 published LUADs^3,4^, *EGFR* ranked second, underscoring its role as a dominant (genetically self-sufficient) LUAD driver (Fig. 3e). We next explored the relevance of this score to clear cell renal cell carcinoma (ccRCC), a tumor that similarly has a recurrent truncal alteration (VHL), comparable mutation burdens, and early diversification (high pITH 67.95%)^35^. In agreement with our hypothesis, VHL too ranked as the most dominant driver in ccRCC (Supplementary Fig. 14), when we applied the same “driver dominance” metric to exome-wide mutation data from the TCGA ccRCC cohort^42^.

These data raise the possibility that early dominant tumor-initiating events can contribute at least in part, to shaping the distinct genomic architecture of tumors. Taken together, our findings from multi-region sequencing of *EGFR*-mutant LUAD suggest that a dominant truncal driver, in the context of low-mutation rates and high-genomic instability, likely results in early clonal selection with subsequent high-intra-tumor heterogeneity.

### Impact of genomic architecture on clinical trajectories

**Fig. 4:**
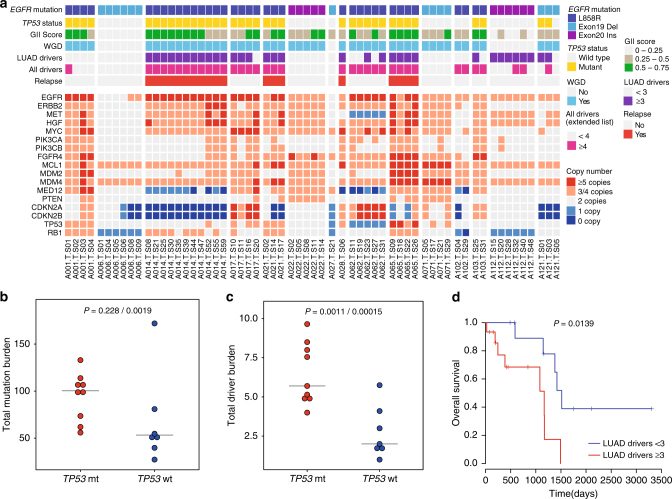
*TP53* mutations, genomic instability, high-driver burden lead to poor outcome. **a** Lower panel is a heatmap representing number of copies for selected genes involved in EGFR TKI resistance or associated with prognosis. Upper panel represents features of a tumor which are associated with patient outcome like *TP53* mutation status, genomic instability index, presence of whole-genome doubling, above and below median number of drivers (LUAD specific or extended driver list) and the relapse status. All these features tend to coincide in many tumors. **b** Total mutation burdens and **c** driver burdens (extended driver list) are compared between *TP53* mutant (mt) and wild-type (wt) tumors. Three random sectors were picked iteratively (*n* = 20) and averages across iterations are represented in **b** and **c**. The first *p*-value is taking all 16 patients into consideration and the second *p*-value is after eliminating the outlier A102 in the analysis. *P*-values are calculated using Welch’s *t*-test. **d** Survival plots using TCGA LUAD *EGFR*-mutant cases (those with non-silent mutations in tyrosine kinase domain, *n* = 26)^4^ after stratifying above or below median number of LUAD drivers (median = 3). *P*-value from *χ*^2^-test is indicated

Out of the five patients who relapsed during the course of this study after surgery (Supplementary Table 1), four carried truncal mutations in *TP53* (A021, A028—both Stage IA; A014—Stage IB; A065—Stage IIA), consistent with its previously reported association with poor outcomes^10,43,44^. The remaining patient who relapsed (A114) had stage IIB disease and prognostically unfavorable clinical features, including lymphovascular invasion, involvement of hilar and intrapulmonary lymph nodes, and was the largest tumor in our series at 6.0 cm (Supplementary Table 1). In addition to the previously described association with WGD and GII (Fig. 2a, b, Supplementary Fig. 12, Fig. 4a, top panel), we found that *TP53*, *EGFR* double-mutant LUAD also harbored higher mutation and driver burdens, both on the trunk and branches (Fig. 4a, top panel, Fig. 4b, c, Supplementary Fig. 15), where out of 9 tumors with ≥3 LUAD specific driver mutations, 8 were *TP53*-mutant. Majority of these drivers were truncal (70.5%, 31/44) although some patients had disproportionately more branch/private mutations, such as patient A121 who harbored a branch *TP53* mutation, and A065 who carried four different LUAD driver mutations in four different sectors (Fig. 1a, c, Supplementary Data 7).

Interestingly, patient A014, who in addition to a *TP53* mutation, carried the highest number of truncal LUAD drivers (five drivers, Fig. 1a, c) and displayed the worst clinical outcome in our series, relapsing in just 4 months. Upon subsequent treatment with gefitinib after relapse, only minor tumor shrinkage was elicited in this patient, with ensuing disease progression and demise within 5 months, consistent with primary TKI resistance. A patient-derived cell line (named 471L cells) from the initial resected primary tumor, was confirmed to harbor the same truncal alterations through targeted re-sequencing (Supplementary Data 8) and similarly exhibited gefitinib resistance (IC_50_ 9.79 µM, as compared to the TKI-sensitive PC-9 cell line, IC_50_ 0.001 µM, Supplementary Fig. 16). Thus, through the clinical course of the patient and patient-derived cell line, we provide functional evidence for the potential role of multiple truncal co-drivers in primary resistance. To validate the impact of number of driver mutations and mutation burdens on outcome, we next examined *EGFR*-mutant patients from the published TCGA cohort^4^. Stratifying patients with respect to number of either driver or all mutations, demonstrated shorter overall survival for cases with higher number of drivers (Fig. 4d; *P* = 0.0139, *χ*^2^-test) or higher overall mutation burdens (Supplementary Fig. 17; *P* = 0.0493, *χ*^2^-test). Taken together, our data suggest that in *EGFR*-mutant LUAD, an early *TP53* mutation may impact clinical outcomes through facilitating genomic instability and the acquisition of additional co-occurring driver events.

On the opposite end of the clinical spectrum, of the seven patients with *TP53* wt tumors, five harbored only the activating *EGFR* mutation as the single-truncal driver. One of these patients, A006 charted an indolent clinical course, having been radiologically diagnosed with ground glass opacities for 5 years prior to surgery. Here, the genomic landscape was distinctly “silent”, with the lowest GII score, no WGD, low-mutation burden and no LUAD co-drivers (Figs. 1a–c, 2a, b,  4a, Supplementary Data 2, 3).

Despite the limited cohort size, these findings illustrate how a spectrum of clinical trajectories might be dictated by the course of genomic events and traits, including *TP53* mutations, presence of multiple truncal drivers, aneuploidy, and associated genomic instability.

## Discussion

Through multi-region sequencing, we have, for the first time, characterized the clonal and subclonal genomic landscape of Asian *EGFR* mutation positive LUAD. Despite the low somatic mutation burden, *EGFR*-mutant LUADs exhibit a heterogeneous genomic landscape characterized by (i) high proportion of late branch and private mutations and (ii) large proportion of genome altered through a combination of early genome doubling events and low-copy gains and losses, followed by late sector-specific copy number changes. Our findings illustrate how timing of genomic events and mutation rates can influence the natural history and diverse clinical trajectories of *EGFR*-mutant LUAD. Founding mutations in *EGFR* and frequent early *TP53* mutations coupled with other truncal alterations deregulating the cell cycle and evading cell death, facilitate tolerance of pervasive WGD and CIN. Despite a relatively high fraction of branch mutations in these treatment naïve tumors, we generally observed a low prevalence of subclonal drivers or putative resistance mutations (e.g., D323N in AKT1), consistent with a neutral evolution model^45^.

While the truncal activating mutations in *EGFR* provide high-response rates to the targeted EGFR TKIs, these responses are often short-lived^27,28^ unlike those to Imatinib in *BCR-ABL1* fusion driven chronic myeloid leukemia (CML)^46,47^. Although the determinants of durability of response in CML remain poorly understood, current studies suggest that the burden of point mutations and SCNAs is moderate in CML compared to solid tumors^48^ which possibly contributes to the longer TKI responses of CML compared to LUAD patients^47^. As a result of ongoing genomic instability in *EGFR*-mutant LUAD, we observed late sector specific copy number amplifications in previously reported genes mediating TKI resistance such as *ERBB2*, *MET*, and *HGF*^25,40^, providing a potential substrate for developing a drug tolerant state. In addition, the subclonal nature of high-amplitude amplifications and deletions underscores the challenge in interpreting gene copy number thresholds e.g., *MET* and *ERBB2*, from single biopsies in NSCLC^49^. Importantly, we implicate the role of multiple co-truncal drivers in a patient exhibiting primary EGFR TKI resistance, with validation in a corresponding patient-derived cell line, and by supporting findings from public data sets. It is thus plausible that co-existing truncal drivers may be associated with a propensity for primary resistance, while minor clones with additional drivers or resistance-mediating alterations can emerge later after initial tumor response to EGFR TKIs. However, larger sample sizes with more functional validations will be needed to test this and to decouple the effects of different genomic features like *TP53* mutations, driver burdens and GII.

**Fig. 5:**
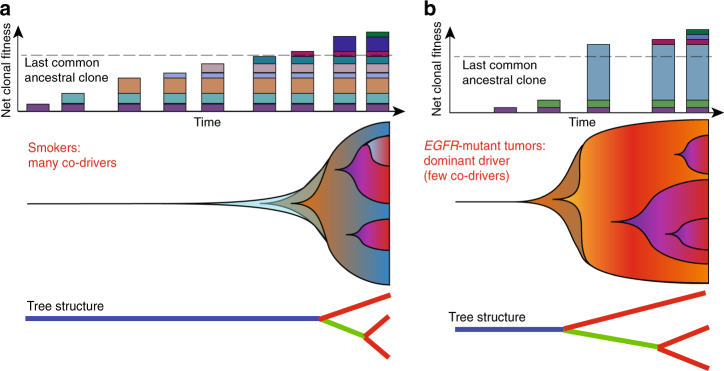
Schematic of evolutionary trajectories in smokers and *EGFR*-mutant non-smoker tumors. **a** In smokers, accumulation of many truncal mutations and drivers before branched evolution. Top: driver mutations are represented as colored rectangles. The dashed line indicates the point of diversification on the tree or the last common ancestral clone (carrying the last truncal driver mutation). Middle: shaded background represents clonal expansion (*y*-axis) as mutations accumulate (*x*-axis) in individual clones (colors). Bottom: schematic representation of phylogenetic mutation tree. **b** The non-smoker scenario where a dominant driver like *EGFR* is hit early leading to a big fitness advantage, fewer clonal sweeps and early diversification. Panel structure as in **a**. In both scenarios, evolutionary trajectory is influenced by clonal dynamics, which in turn is related to competitive fitness of the individual cell populations. Example of such factors include but are not limited to mutations rates, driver nature, cytokine milieu, immune cell infiltration, and metabolic conditions

An unexpected observation was the high pITH in never-smoker-enriched *EGFR*-mutant LUAD with comparable branch/private mutation burden to smoker LUADs. Although our study does not allow delineation of all the intermediate steps and selective sweeps preceding the final clonal composition, it nevertheless illustrates the contrasting life histories and a distinct evolutionary trajectory of *EGFR*-mutant LUAD, compared to smoking-related LUADs^19,20^. In the smoking scenario, a tumor-initiating cell population acquires mutations at a high rate and hence accumulates a large number of passenger and relatively weak driver mutations, with low likelihood for early acquisition of dominant drivers like *EGFR* (Fig. 5a). On the background of high-mutation rates, it is possible that, consecutive drivers are gained within a time frame that results in selective clonal sweeps with eventual population expansion and long trunks as previously observed (Fig. 5a). In contrast, in an oncogene-driven never-smoker LUAD, a dominant driver e.g., *EGFR* mutation, in the context of low-mutation rates—is sufficient to allow expansion of early tumor cells with few co-drivers. As a result of low-mutation rates, subsequent drivers occur late and are less likely to cause complete sweeps, resulting in shorter trunks and early diversification (Fig. 5b). Given the inter-patient differences in genomic architecture in our current series, further studies are required to unravel the additional determinants of clonal dynamics for each patient, such as the cytokine milieu^50^, immune cell infiltration^51^ and differential metabolic phenotypes in the tumor microenvironment^52^; as well as how these may relate to selective pressures imposed by anticancer therapies.

In summary, we have elucidated the distinct clonal architecture of *EGFR* mutation positive LUAD, providing insights as to how these may relate to the diverse clinical trajectories observed. While dominant truncal drivers, such as *EGFR* mutations are an important prerequisite for efficacious targeted therapies, the evolutionary trajectory for each tumor can be augmented by additional genomic events in the natural life history, enhancing clonal fitness with emergent drug resistance. Finally, we suggest that co-occurring truncal drivers and extent of genomic instability can have potential clinical value as biomarkers for risk stratification. Comprehensive depiction of the genomic landscape of *EGFR*-mutant LUAD may offer opportunities for development of high-precision therapeutic strategies tailored to individual risk of disease progression.

## Methods

### Patient cohort and sample processing

Among the patients diagnosed with LUAD at the National Cancer Centre Singapore, which underwent surgical resection of their tumors prior to receiving any form of therapy, 16 patients carrying *EGFR* mutations were selected for this study (relevant clinical information of each patient is provided in Supplementary Table 1). Written informed consent was obtained from all participating patients. The study was approved by the relevant Institutional Review Board (Singhealth Centralised IRB, Singapore).

Resected tumors were sectioned horizontally and tumor tissue in each section was cut into four quadrants (A–D; Supplementary Fig. 1), which if large enough were processed further into smaller sectors. Pathologists’ evaluated sectors for reasonably high-tumor content were snap frozen for DNA/RNA sequencing. Neighboring horizontal sections were used for histological analyses. Adjacent normal lung tissue or blood was used as a matched normal control. DNA and total RNA extractions were performed from frozen tissues using Qiagen All prep universal kit and the DNA was subjected to library preparations for sequencing as described below.

### Whole-exome sequencing

Quantity of 500 ng to 1 µg of genomic DNA was sheared using Covaris to a size of 300–400 bp and subjected to library preparation using NEBnext End repair, A-tailing and Ligation modules (New England Biolabs). 3–6 samples were pooled together and hybridized using the SeqCap EZ Human Exome Library v3.0 (Nimblegen, Roche) kit. Captured regions were washed, purified, amplified, and subjected to 2 × 101 sequencing on the Hiseq 2000 to obtain a mean coverage of 114X.

### Targeted amplicon deep sequencing

Primers were designed around the somatic variants (SNVs and Indels) annotated to be in the exonic and splice regions, using the Generead DNA-seq custom panel v2 (Qiagen). In addition, primers covering the T790M locus in *EGFR* were also added to the panel. Amplicons were generated according to manufacturer’s recommendation and libraries were prepared using the NEBnext Ultra DNA-seq kit (New England Biolabs). Libraries were then pooled and sequenced on Hiseq 2500 by 150 bp paired end reads to obtain a mean depth of 3860X.

### SNV and indel calling from exome-seq

Reads were mapped to the human reference sequence GRCh37 (hg19) using the bwa-aln algorithm^53^ using default parameters. Duplicate reads were marked using Picard tools after which realignment around known indels and base quality recalibration was performed at an individual sample level using GATK 2.7 version^54^.

Somatic mutation calling was performed using MuTect^55^ allowing up to 5 reads supporting the variant allele in the normal sample up to a maximum of 0.05 allele frequency. The passed variants were further filtered using the described criteria to obtain a more confident set of somatic variants. A minimum of 6 reads supporting variant allele in the tumor was mandatory. To improve accuracy in low-frequency calls, variants with allele frequency (VAF) in tumor below 0.2 were treated as somatic only if the tumor allele frequency was at least 10 times greater than normal allele frequency for the variant allele.

To eliminate false positive variant calls due to polymerase chain reaction (PCR)-chimeras formed in exome-seq protocols, germline variants were called from the normal sample of all patients as described below and were filtered out from the somatic variant list.

Somatic indels were called using Strelka^56^ and were further filtered for >4 reads (MAPQ >20) supporting the indel with a minimum allele frequency of 0.1.

The final list of somatic SNVs and indels was then annotated by multiple databases using the Annovar tool^57^.

### Germline variant calling from exome-seq

Germline variants for each patient were called from the normal sample using GATK Unified Genotyper after indel realignment and recalibration of bam files^54^. Variants were then subjected to hard filtering using GATK recommendations to obtain a more confident set of SNVs and indels.

### Variant calling from targeted deep-seq

Fastq files were mapped to the human reference sequence GRCh37 (hg19) using bwa-mem algorithm with default parameters. The bam files were realigned and recalibrated using GATK. Since the amplicon size is around 150–200 bp, the reads obtained were overlapping. For each base at the overlapping region, if the pair bases were identical, quality score for both bases were updated to original quality score ×1.2; else if the pair bases were not identical, the base with the lower quality score was replaced by the base with the higher quality score, and both quality scores were updated to original higher quality scores ×0.8.

Somatic SNVs were then called using VarScan v2.3.7^58^. Default parameters were used except the minimum variant frequency was set to 0.01. Only those variants also called in the exome-seq were considered. Variants with <10 reads supporting the alternate allele were filtered out and variants with allele frequency below 0.05 were mandated to have a minimum alternate read count of 15. Further, mutation calls were required to have a VAF five times higher in the tumor compared to the normal.

For somatic indels, read counts supporting the indel identified by exome-seq for that patient were obtained from both the tumor and normal bam files using a custom script. Reads properly paired and mapped with MAPQ >20 were counted. Somatic indels with >90% read counts from one strand were removed from further analyses. Further only those indels were treated as validated which had at least 10 reads supporting the alternate allele at a frequency five times greater in tumor than in normal with a minimum VAF of 0.03 in the tumor.

A validation rate of 94% for indels and 85% for SNVs was achieved for the exome-seq data. Only validated variants were considered for generation of phylogenetic trees and any downstream analysis.

### Phylogenetic analysis

Using the presence and absence of somatic mutations across samples, we first calculated the genetic distances between samples using the hamming distance. The neighbor joining algorithm from the APE package^59^ was used to infer phylogenetic relationships between tumor sectors for each patient.

### Comparison with published data

The published LUAD data sets^19,20^ were retrieved from European Genome-phenome Archive (EGA). The corresponding EGA data set-IDs are: EGAS00001000930 and EGAD00001000900. In order to directly compare the pattern with our data set, we selected only those patients, where the tumor was restricted to a single site and had pure adenocarcinoma histology. The downloaded targeted deep sequencing data were processed using the same pipeline as our Singapore cohort data. Since the fraction of trunk mutations in the phylogenetic trees is a function of the number of sectors, we calibrated the trunk ratio by performing random subsampling of sectors for each patient. The average proportion of the trunk was compared across cases conditioning on the same number of sectors.

### Mutation signatures analysis

In order to uncover mutational processes active within the EGFR mutant LUAD patients, we combined somatic mutations from the Singapore cohort with two published large-scale data sets^3,4^. With the information from the point mutation and the flanking 5′ and 3′ bases, the Emu package^60^ was used to infer the mutation signatures in the 79 tumor sectors.

### Driver genes annotation

We defined LUAD driver genes (*n* = 78) through significantly mutated genes in LUAD collected from seven publications^1–4,61–63^. Mutations in these genes are shown to occur more than just by chance or due to the size of gene. Other cancer driver genes include additional significantly mutated genes in at least one cancer type from two pan cancer studies^62,63^ and remaining genes in cancer gene census^64^ (*n* = 735). Non-silent mutations in these two lists of driver genes were annotated on the trees. For comparison across data sets, either LUAD genes or combined set of both groups of drivers (extended driver list) was used as indicated in main text or figure legends.

### Copy number analysis

Illumina omniexpress arrays were run using DNA from all 79 tumor sectors along with matched normal tissue using protocols suggested by the manufacturer. Log ratio (LRR) and B allele frequency (BAF) for all SNPs on the array were obtained from Genome Studio. These LRR and BAF values were used as input for ASCAT^65^ v2.4.1 along with the gender information. GC correction, followed by segmentation and purity/ploidy predictions along with obtaining allele-specific integer copy numbers was performed using ASCAT^65^. Each solution was manually checked and samples for which ASCAT could not provide a reliable solution (e.g., purity = 1) were eliminated from further analysis. 61 out of 79 samples from 15 patients remained after these filtering. For 5 of these 61 samples (namely A001-T-S03, A017-T-S10, A021-T-S02, A112-T-S28, and A112-T-S40), ASCAT solution was manually picked using second or third most optimal purity/ploidy solution since either the raw data suggested these sectors to be similar to other sectors from same patient or variant allele frequencies suggested alternate purity solution. While presence of multiple clones within a sector might lead to such alternate solutions in some scenarios^18,65^, we chose to be conservative in absence of a gold standard and removed any potentially artificial heterogeneity. Copy number losses or gains were determined relative to the median integer ploidy of the tumor which was obtained as the median integer copy number of the SNPs used in ASCAT analysis.

Genomic Instability Index (GII) was calculated as the fraction of the total genome which was altered by any copy number gains or losses with copy change ≥1 defined relative to median integer ploidy. adGII (amplification and deletion based genomic instability index) scores were calculated as fraction of genome affected by high-copy gains and losses (or amplification and deletions with copy change ≥2 relative to ploidy). To obtain cytoband or gene level copy numbers, chromosomal locations of cytobands and genes were overlapped with those of the segments and the segment copy number was assigned to that cytoband or gene. In case multiple segments overlapped with the cytoband/gene, a minimum of 25% overlap was made mandatory and the segment with the highest overlap was used to assign the copy number to the cytoband/gene. Known driver regions in LUAD from previous large scale studies^4,6–8,41^ were curated and only those regions were considered which were altered in same direction in at least two studies.

### Genome doubling status

The genome doubling status for each tumor sample was determined using a published algorithm^38^. In brief, a *p*-value was obtained using 10,000 simulations with observed probabilities of copy number events. For samples with ploidy ≤3, a *p*-value threshold of 0.001 was used. To avoid underestimating genome doubling in high-ploidy samples, a *p*-value threshold of 0.05 was used for samples with ploidy = 4, and all samples were classified as genome doubled if the ploidy exceeds 4.

### Timing of mutations relative to copy number or genome doubling

The cancer cell fraction (CCF) and mutant allele copy number for a given SNV was calculated following the algorithm described previously^16^, where the corresponding integer copy number and tumor purity were derived using SNP-array and ASCAT algorithm. A given mutation was classified as “clonal” if the 95% confidence interval of CCF overlapped 1, and “subclonal” otherwise. The timing of a given mutation relative to copy number alteration was classified on its clonal status and the integer rounded mutant allele copy number. Indels, regions with no copy number alteration (major copy number = minor copy number = 1), regions with just single copy were eliminated as these could not be evaluated for timing. Mutations were called early only when (i) the mutation is clonal, and (ii) the rounded integer mutant allele copy number ≥2.

### Dominance of the driver genes

The dominance of a driver (driver self-sufficiency) was calculated for all known LUAD drivers (*n* = 69) and ccRCC drivers (*n* = 16) which were found mutated in ≥5 patients in the TCGA data. This was based on the logic that, for each cancer patient, the number of driver mutations found in each case implies the self-sufficiency of the drivers. Mutations which possess high capability to drive tumorigenesis would exist with few co-occurring driver mutations since these will be the sufficient to initiate a tumor. Using the combined public data sets^3,4^, we computed a measure for driver dominance of each gene as

$$D_{\rm i} = \frac{{\mathop {\sum }\nolimits_{j = 1}^{N_{\rm i}} 1/d_j}}{{N_{\rm i}}},$$

where *D*_i_ is the dominance score for gene *i*, *N*_i_ is the number of patients carrying non-silent mutations in gene *i* while *d*_*j*_ is the number of LUAD driver mutations in patient *j*.

Therefore, the genetic dominance (i.e., self-sufficiency) (*D*_i_) we define is inversely proportional to the number of co-occurring driver mutations across samples.

ccRCC drivers were picked from the TCGA publication as significantly mutated genes^42^.

### Cell culture

471L cells were derived from second generation of xenograft from A014 tumor (cells from first xenograft were transplanted into a second mouse). The tumor was collected, digested with 1 mg/ml Collagenase IV (Gibco, Life Technologies) and cultured on 100 mm plastic dish with culture conditions similar to those described earlier for patient-derived cell lines^24^. Using targeted sequencing we had confirmed that mutation found in the cell lines matched with the primary patient tumor (Supplementary Data 8).

PC9-GefR cell line was generated by exposing PC9 cells (a gift from Dr. Sin Tiong Ong, Duke-NUS University, Singapore) to stepwise increment doses of Gefitinib (0.1–6.4 µM). Both isogenic cells were maintained in RPMI (Sigma-Aldrich), supplemented with 10% FBS (Hyclone, Fisher Scientific), 100 units/ml of Penicillin, 100 µg/ml of Streptomycin, and 0.25 µg/ml of Amphotericin B (Antibiotic-Antimycotic Gibco, Thermo Fisher Scientific).

All cell lines were cultured in clean, well established cell culture labs with no sort of mycoplasma or other contamination.

### Dose response to Gefitinib

471L, PC9 and PC9-GefR cells were seeded into 96-well plates and were treated with varying concentration of drugs the next day (0.0001–20 µM). CellTiter-Glo Reagent (Promega) was added directly to the cells per manufacturer’s instructions after a 72 h treatment period. Luminescence was measured to determine the amount of viable cells. Percentage cell viability was calculated relative to 0.2% DMSO vehicle control. All cell viability assays were performed in triplicates. Dose response curves were generated using GraphPad Prism version 7.

### Data availability

All the sequencing and SNP array data have been deposited at the European Genome-phenome Archive (EGA, http://www.ebi.ac.uk/ega/), which is hosted by the EBI, under the accession code EGAS00001001736.

## Electronic supplementary material

Supplementary Information

Description of Additional Supplementary Files

Supplementary Data 1

Supplementary Data 2

Supplementary Data 3

Supplementary Data 4

Supplementary Data 5

Supplementary Data 6

Supplementary Data 7

Supplementary Data 8
