# Supplementary material for: Elucidating the genomic architecture of Asian EGFR-mutant lung adenocarcinoma through multi-region exome sequencing
Source: Nat Commun. 2018 Jan 15;9:216. doi: 10.1038/s41467-017-02584-z (PMC5768770; doi:10.1038/s41467-017-02584-z)
Supplement: Supplementary file 1 — Supplementary Information [file 41467_2017_2584_MOESM1_ESM.pdf]

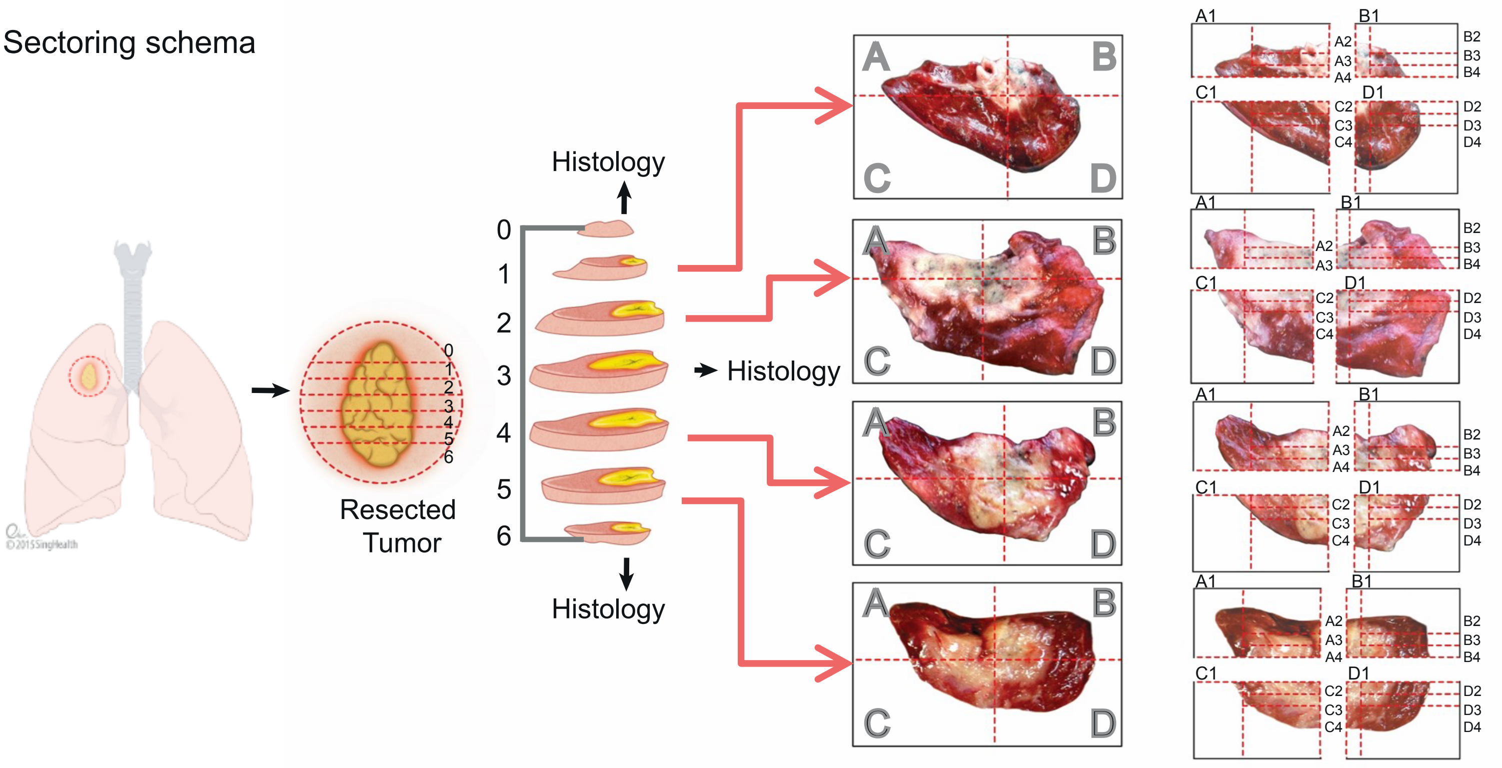

Supplementary Figure 1. Sectoring Schema. Diagram representing the sectoring protocol for the resected lung tumors. Depending on the size of the tumor, it was first horizontally sectioned and some sections with reasonable tumor content were cut further into 4 quadrants. The quadrants depending on the size were further dissected into smaller portions or sectors to obtain approximately >=20 mg tumor sectors. These sectors were snap frozen for nucleic acid extractions. Every tumor section used for genomic studies had a section next to it preserved for histological studies. The drawn diagrams are reproduced here with permission from SingHealth Academy. The photographed resected tumor section images were taken by co-author Angela Takano for use in this paper.

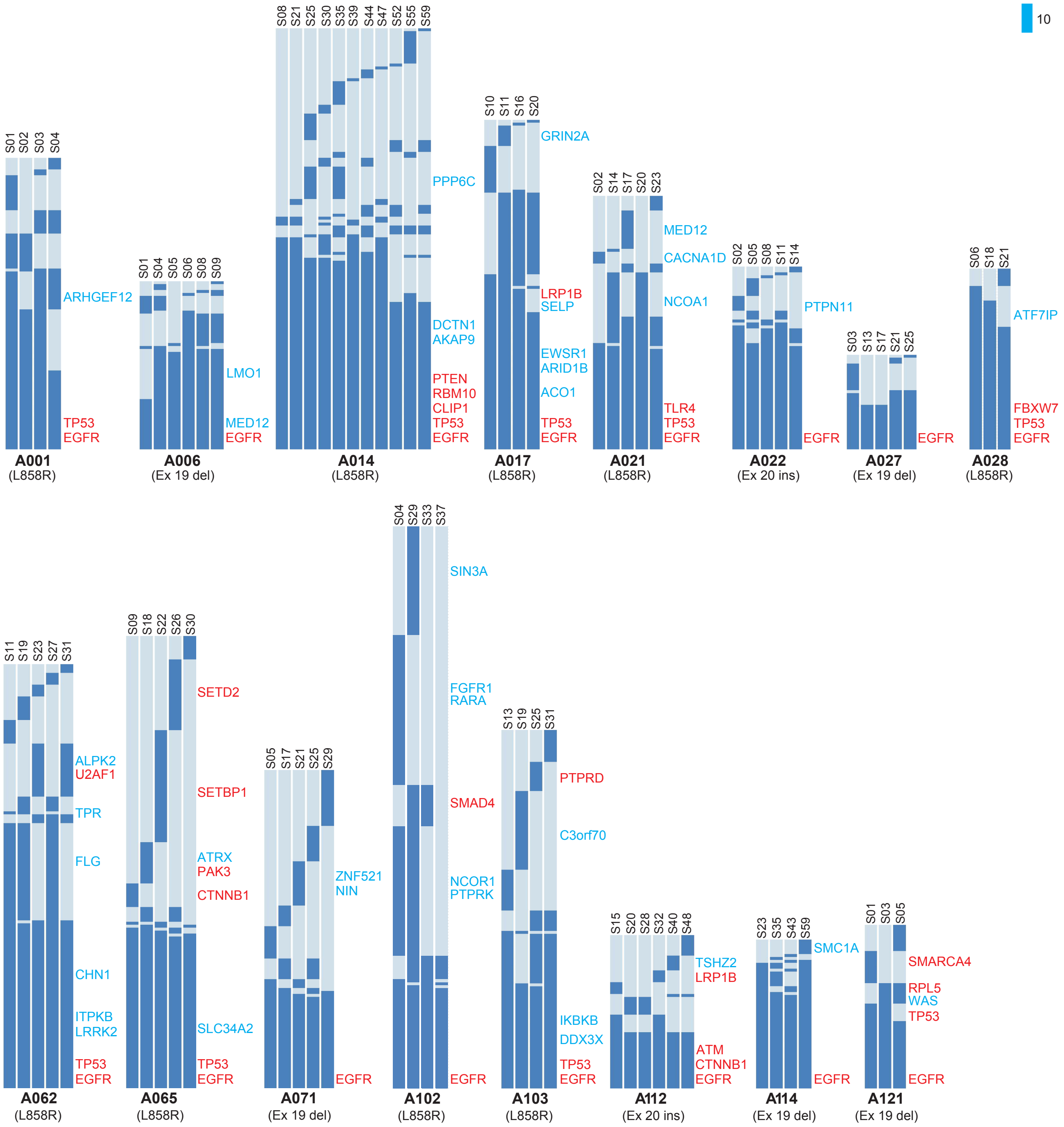

Supplementary Figure 2. Heatmap view to represent ITH in Asian *EGFR*-mutant LUAD tumors. Darker shade of blue indicates presence of mutations while lighter shade indicates absence. The height of the heatmaps are proportional to the total number of mutations. Scale bar is on the top right corner. Non-silent LUAD-specific driver mutations are in red while other cancer drivers are shown in blue.

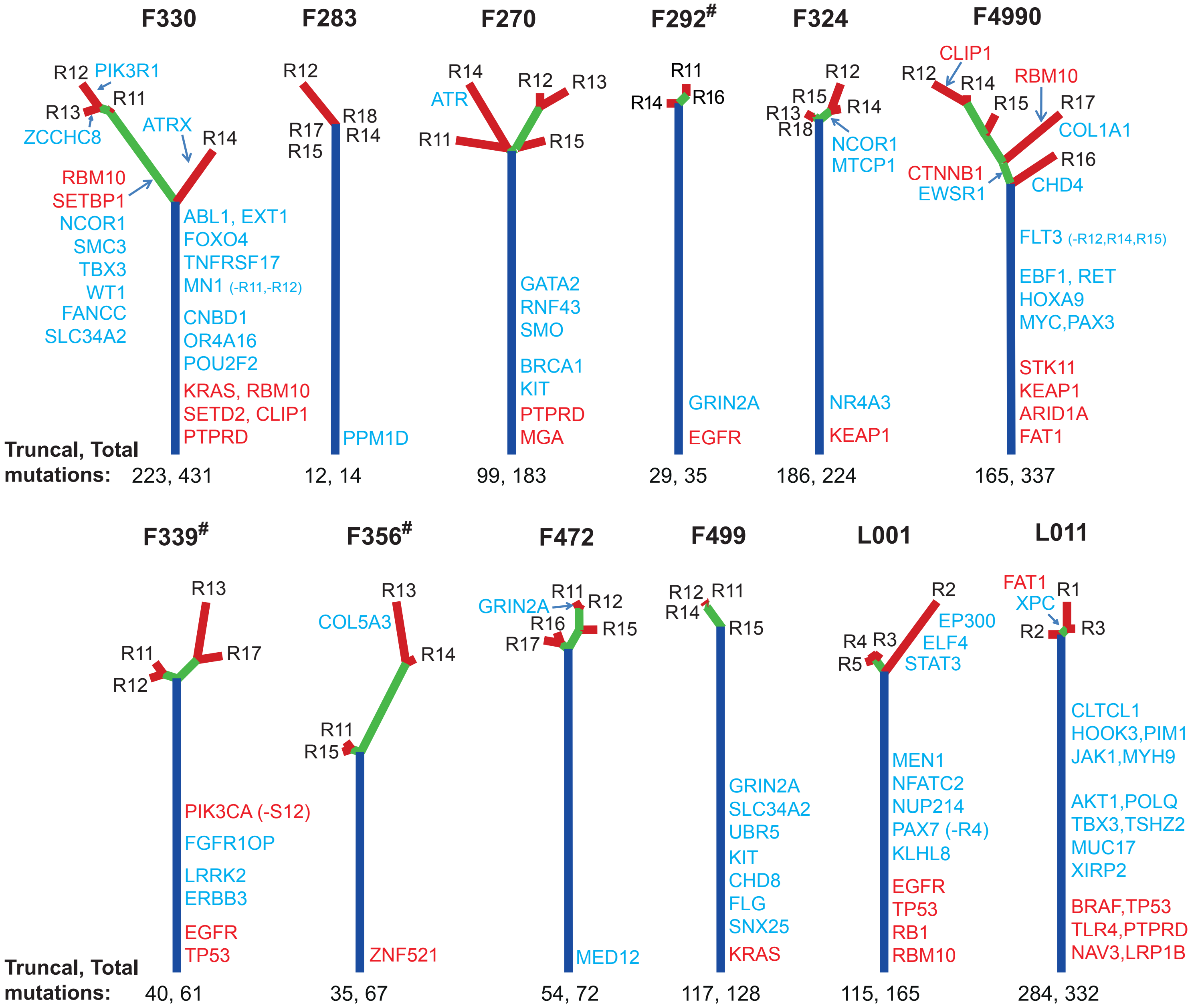

Supplementary Figure 3. Phylogenetic trees for published Caucasian samples. Targeted deep sequencing data on variants identified from multi-region exome-seq done on 12 tumors from the two published studies on Caucasian smoker dominated LUAD cases[1,2] was analysed through our pipelines. Phylogenetic trees were generated in the same way as our *EGFR*-mutant samples. All trees are adjusted to the same height. LUAD specific known drivers are annotated in red and other cancer drivers in blue. Patient IDs marked with a '#' are non-smokers.

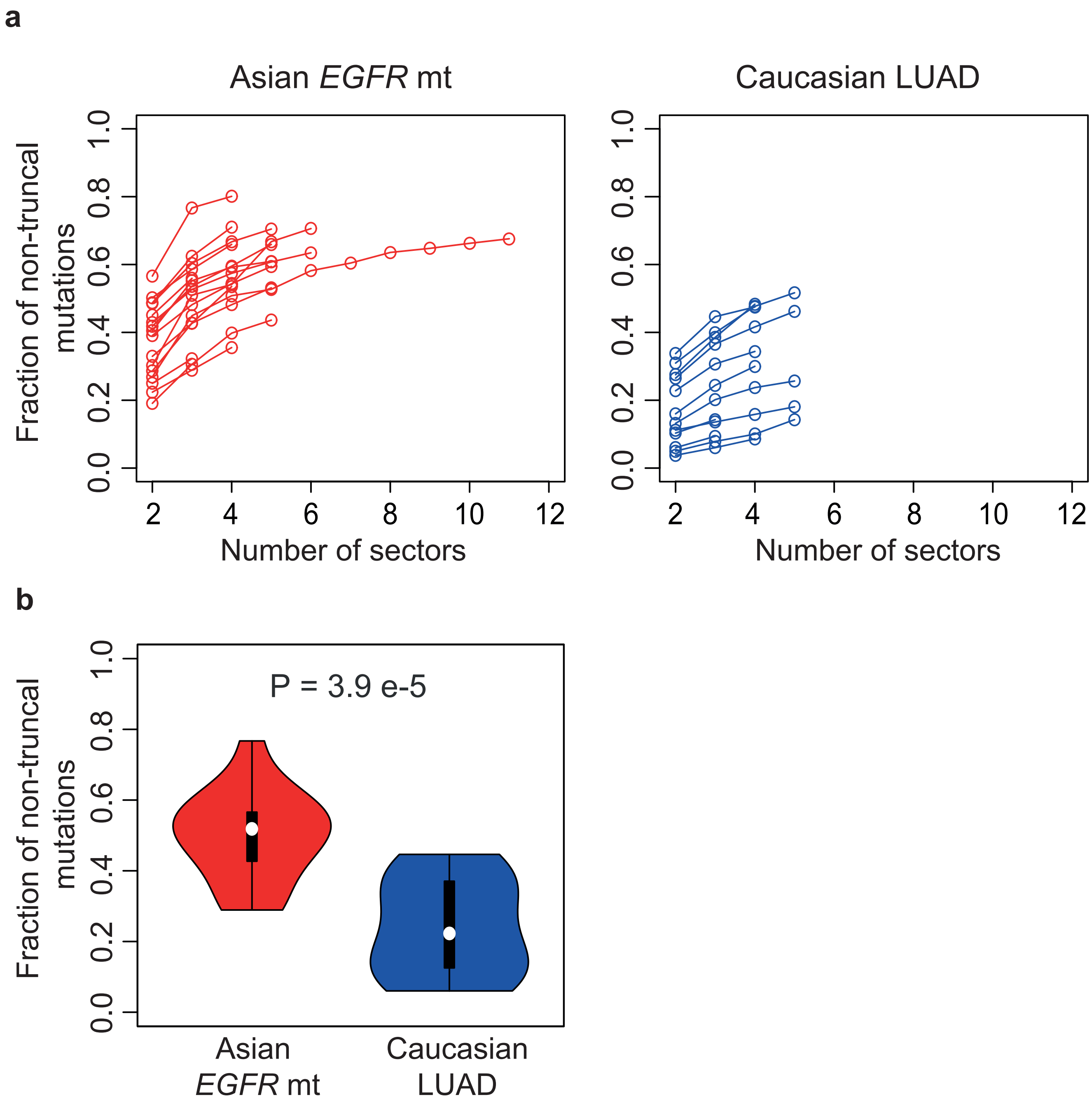

Supplementary Figure 4. Asian *EGFR*-mutant tumors have higher ITH. (**a**) Fraction of non-truncal mutations (pITH) after random sampling of different number of sectors for Asian *EGFR*-mutant tumors (left) or the published smoker dominated Caucasian tumors (right)[1,2]. (**b**) Fraction of non-truncal mutations (pITH) after randomly picking three sectors is significantly higher in our *EGFR*-mutant tumors compared to the published data on smoker dominated Caucasian LUAD (P= 3.9 x 10^{-5}, Welch's t-test). White circle within the black box indicates the median.

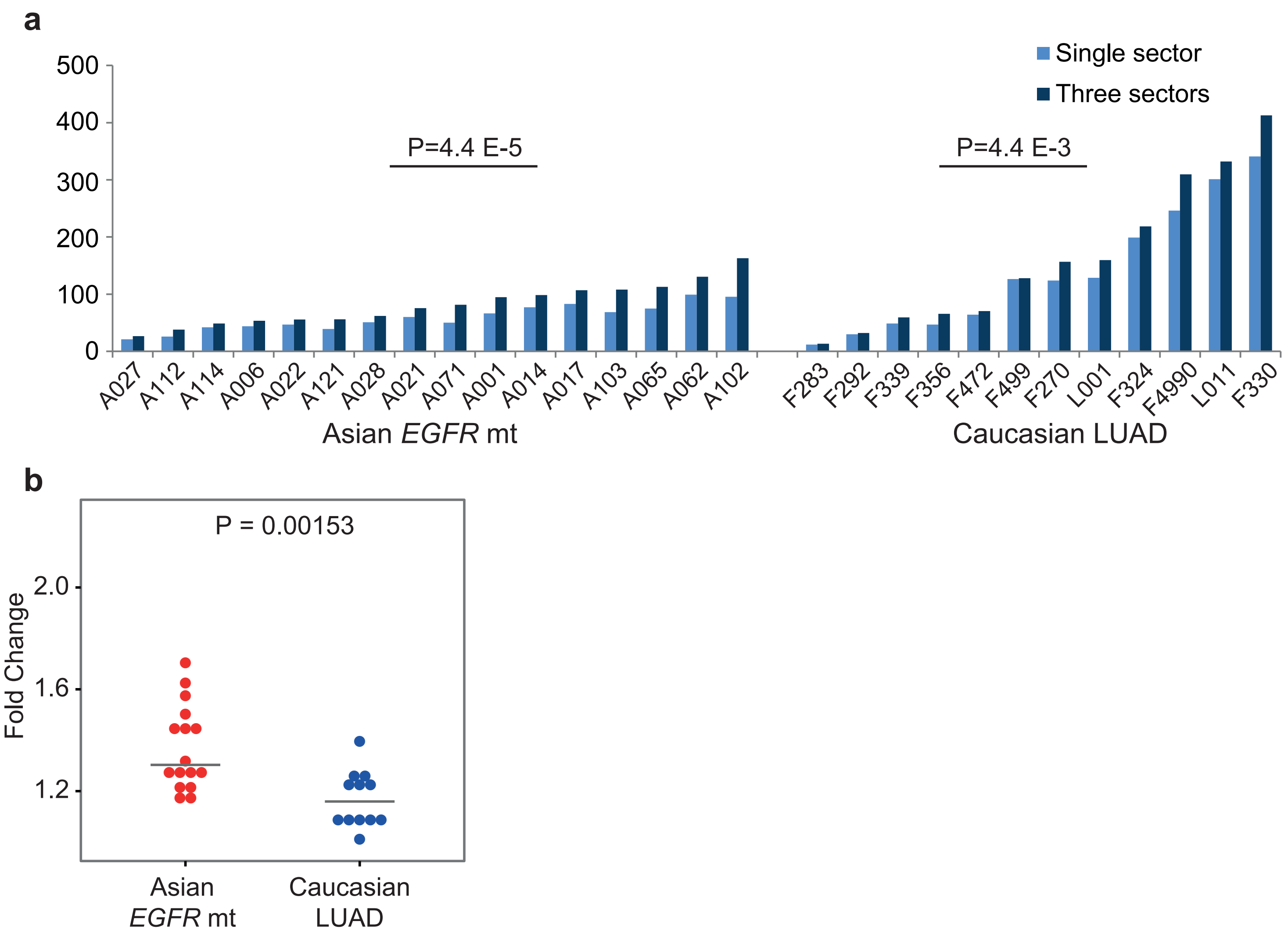

Supplementary Figure 5. Multi-sector sequencing reveals mutations invisible by single sector sequencing. (**a**) Bar plots showing significant increase of detected mutations upon multi-sector sequencing compared to single sector sequencing in both cohorts. P-values from paired t-tests are indicated. Single sector mutation burden (number of detected mutations) represents the median mutation burden across individual sectors of any tumor while multi-sector mutation burden represents mean mutation burden upon random selection of three sectors. (**b**) The fold increase in mutation burden upon multi-sector sequencing was significantly higher in Asian *EGFR*-mutant LUAD enriched for non-smokers compared to smoker-enriched Caucasian LUADs (P=0.00153, welch's t-test). The horizontal line indicates the median for the cohort.

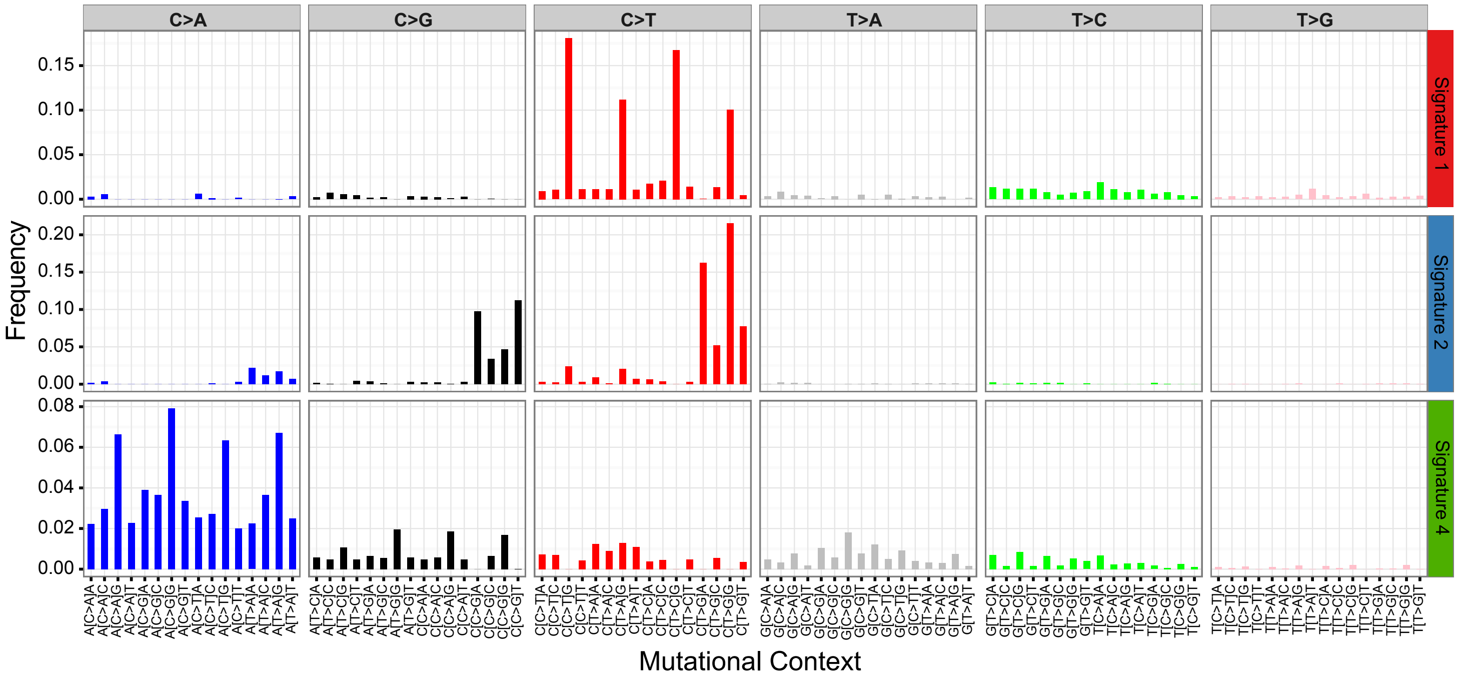

Supplementary Figure 6. Mutation signatures. Three mutation signatures were identified in the 16 *EGFR*-mutant LUAD. Signature numbers are according to the COSMIC nomenclature.

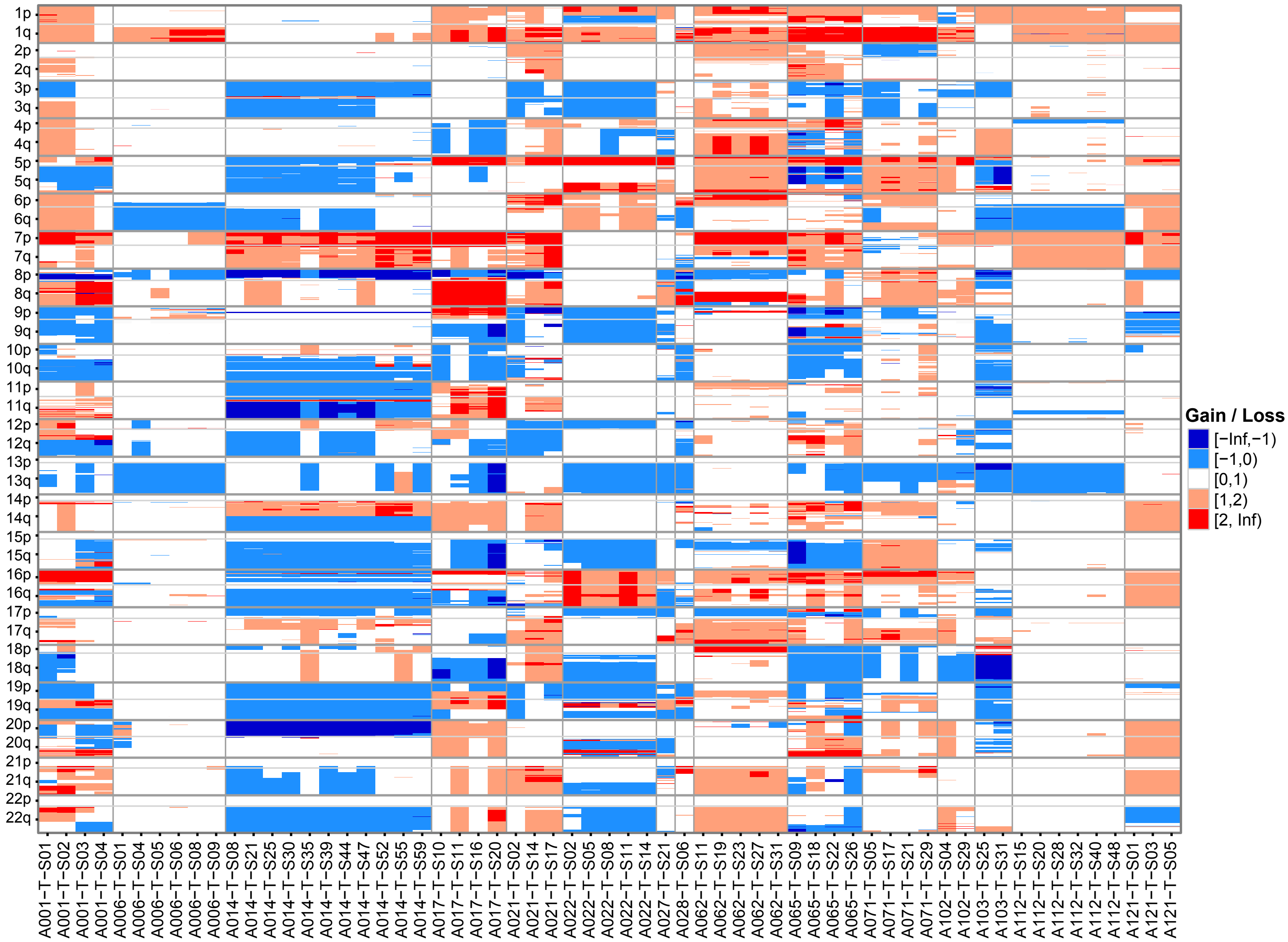

Supplementary Figure 7. Genome-wide copy number landscape. Somatic copy number landscape evaluated relative to ploidy of individual tumors. Gains of single copies are in light red while those of two or more copies beyond ploidy are in dark red. Losses of single copies and two or more copies relative to ploidy are depicted in blue and dark blue respectively.

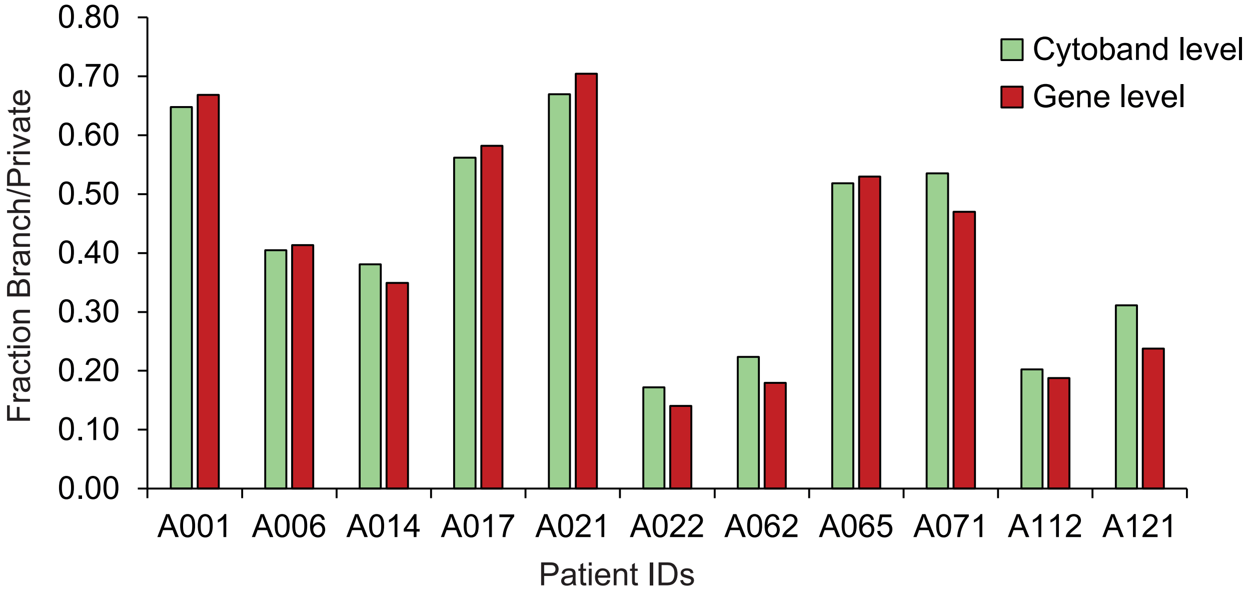

Supplementary Figure 8. Proportion of branch/private copy number alterations. For tumors with >=3 evaluable sectors, fraction of cytobands and genes with non-truncal copy number alterations are represented after random selection of three sectors. Presence or absence of amplifications and deletions across sectors is considered regardless of degree of copy change relative to ploidy (any region with >=1 copy change included).

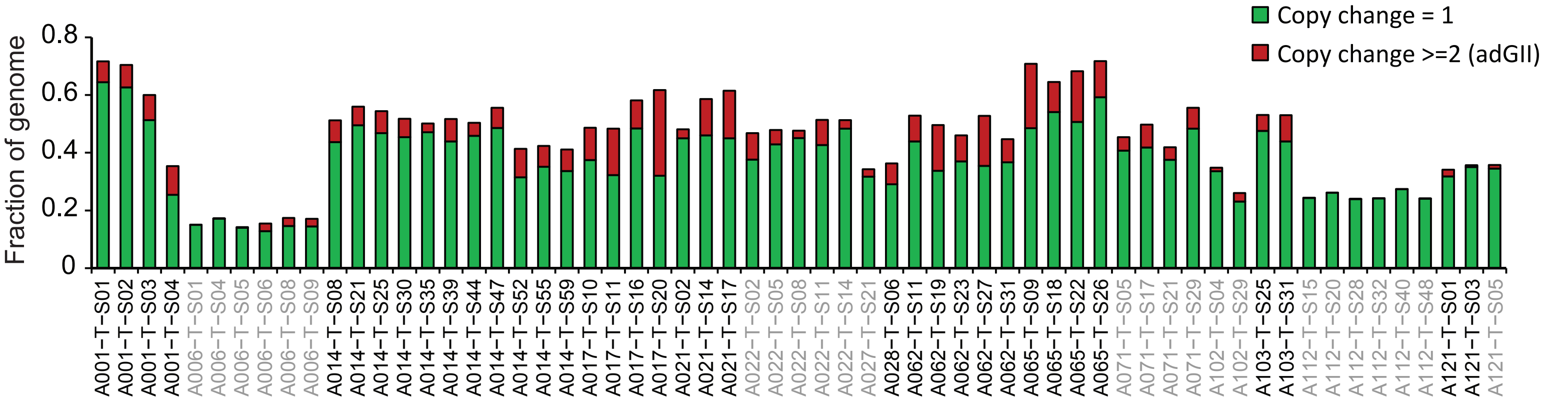

Supplementary Figure 9. Fraction of genome affected by copy number alterations. Fraction of genome affected by low copy gains and losses (copy change =1 relative to ploidy) are shown in green and fraction affected by high copy amplifications and deletions (copy change >=2 relative to ploidy) is shown in red. The sum of both gives GII scores. Tumor samples labelled in grey are *TP53* wildtype.

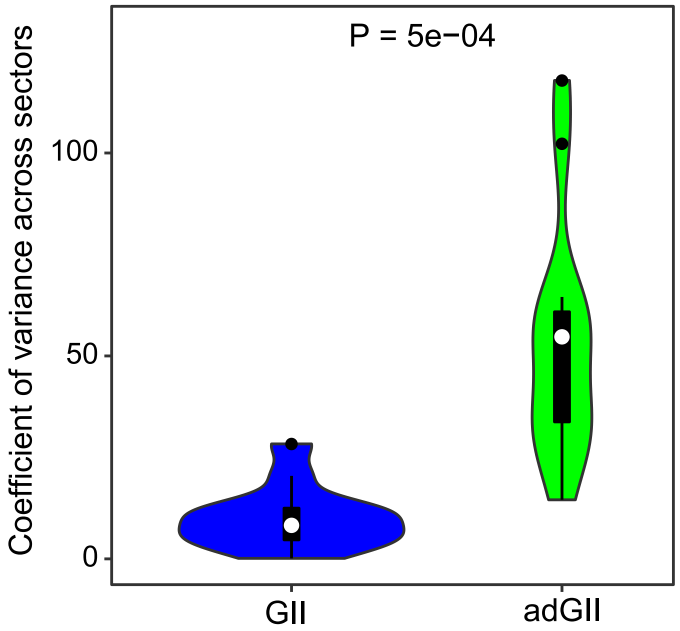

Supplementary Figure 10. Higher variance across sectors for adGII scores compared to GII. Genomic instability index (GII) calculated as fraction of genome affected by any SCNA (copy change >=1 relative to ploidy) had lower variance across sectors of a tumor as they are dominated by low copy gains and losses (Supplementary figure 9). adGII or genomic instability index due to amplifications and deleitons which is calculated as fraction of genome affected by high copy amplifications and deletions (copy change >=2 relative to ploidy) showed higher variance across sectors of a tumor suggesting that gain in amplitude of SCNAs are late events. The white circle indicates the median coefficient of variance for the respective cohorts. P-value from Welch’s t-test is indicated.

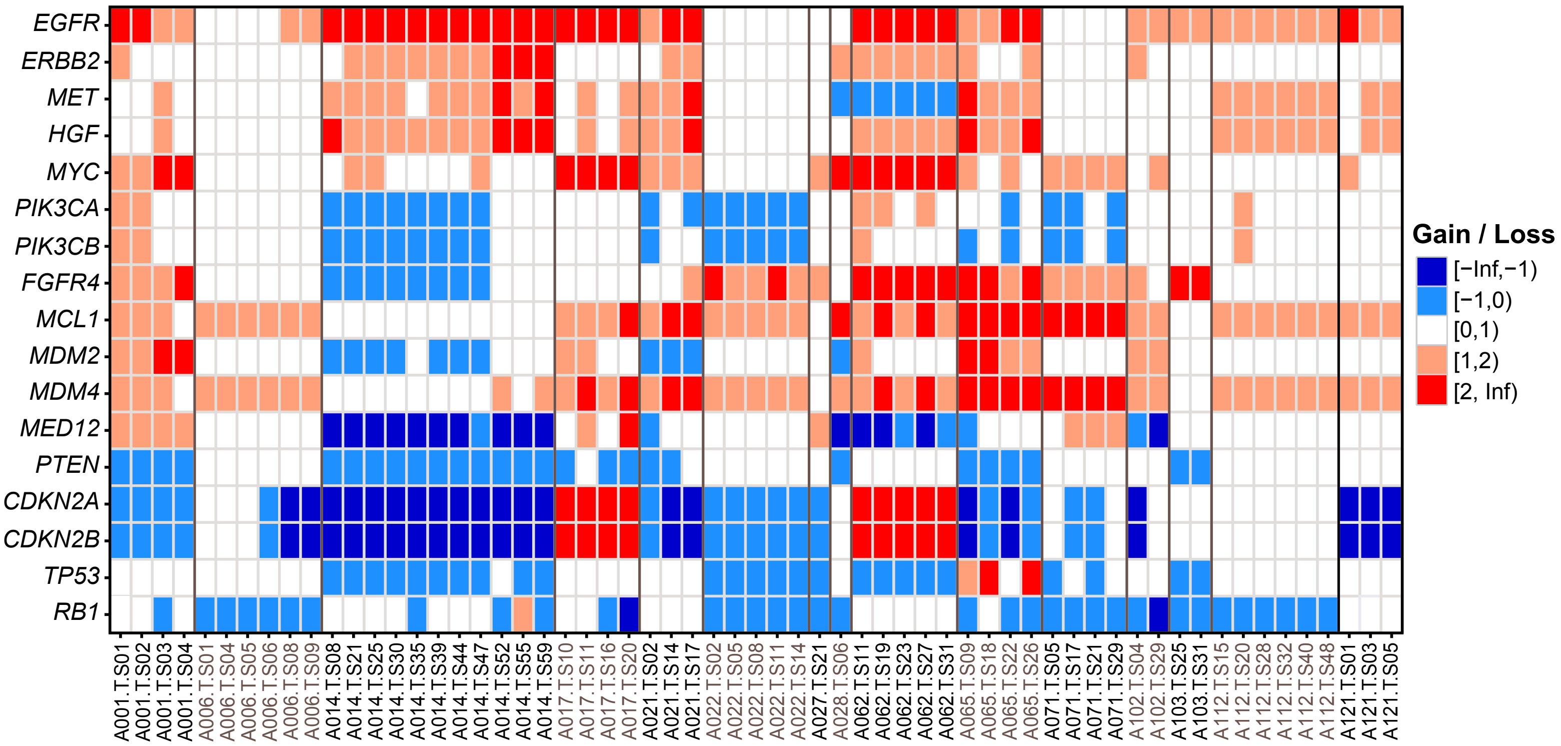

Supplementary Figure 11. SCNAs in resistance and tumorigenesis relevant genes. Heatmap representing copy number gains (red) and losses (blue) for genes shown relative to ploidy of the tumor sector. Lighter shades indicate gains or losses of one copy while darker shades show relative gains and losses of >=2 copies. Genes were selected based on well established involvment in EGFR TKI resistance and tumorigenesis in cancer literature. Tumor samples labelled in grey are *TP53* wild-type.

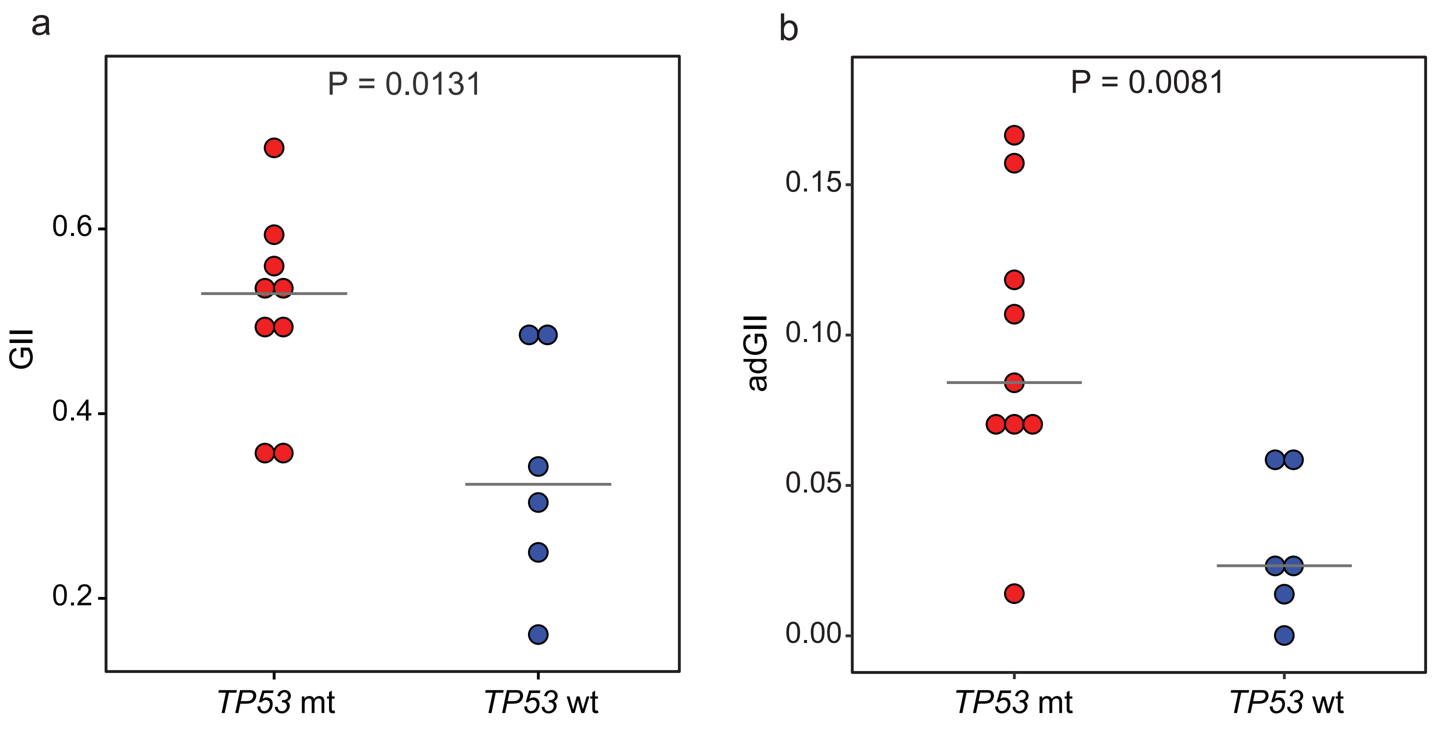

Supplementary Figure 12. Higher genomic instability in *TP53* mutant tumors.
Comparison of (**a**) overall genomic instability index (GII) and (**b**) amplification and deletion based genomic instablity index (adGII) between *TP53* wild-type (wt) and mutant (mt) groups. Average GII and adGII scores across sectors from same patient are compared using Welch’s t-test. Horizontal lines indicate the respective median scores.

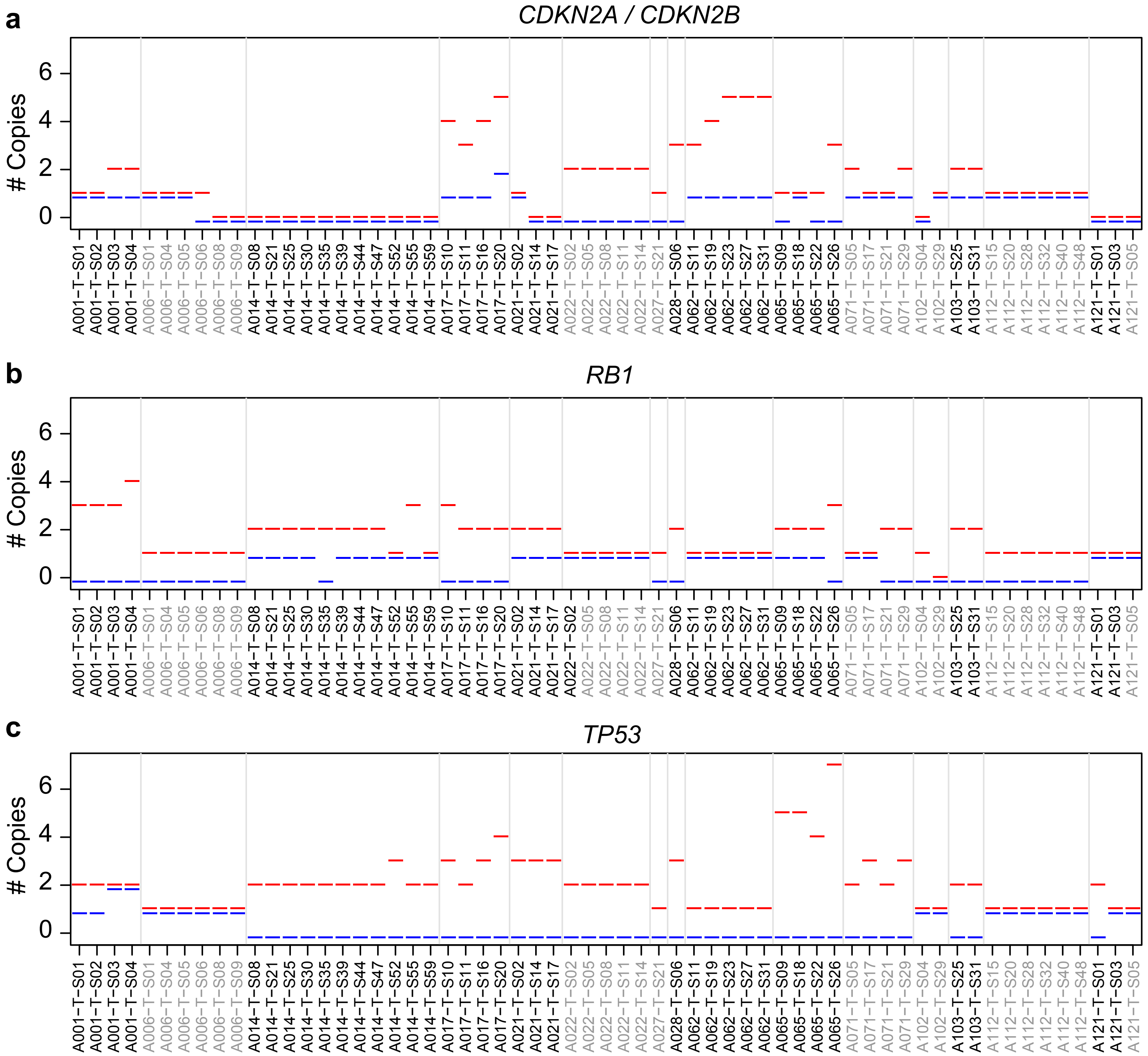

Supplementary Figure 13. Allele specific copy numbers. (**a**) *CDKN2A*, *CDKN2B*, (**b**) *RB1* and (**c**) *TP53* genes were mapped on the ASCAT predicted copy number segments and corresponding allele specific integer copy numbers were assigned to these regions of interest (y-axis). Red and blue lines represent the major and minor allele respectively. Grey shaded tumor samples are *TP53* wildtype. LOH events were identified as those with zero copies for minor allele but atleast one copy for major allele.

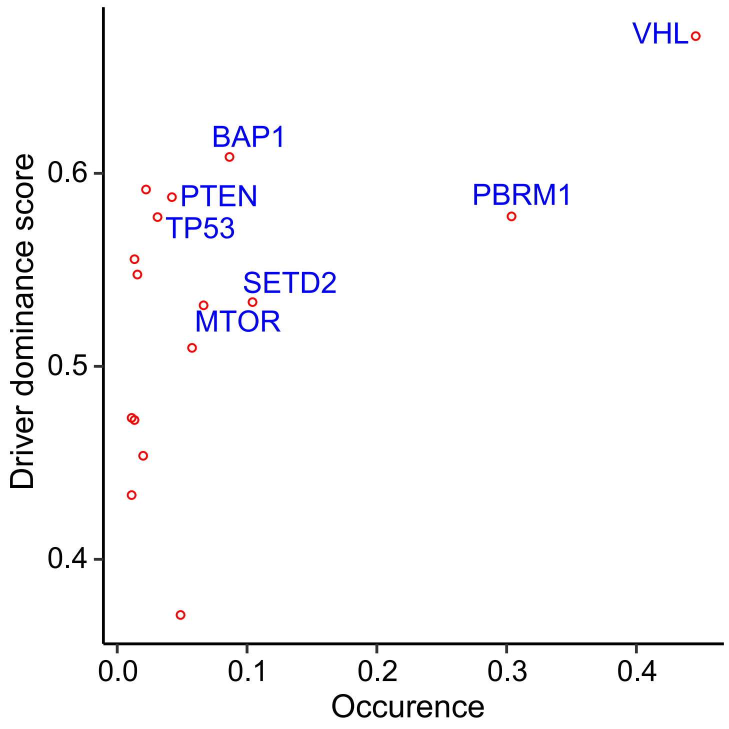

Supplementary Figure 14. ***VHL*** **is a dominant driver in ccRCC.** Our driver dominance metric was applied to 16 drivers occuring in >=5 patients in the ccRCC TCGA cohort[3] and we identified the recurrent truncal *VHL* gene to rank the highest.

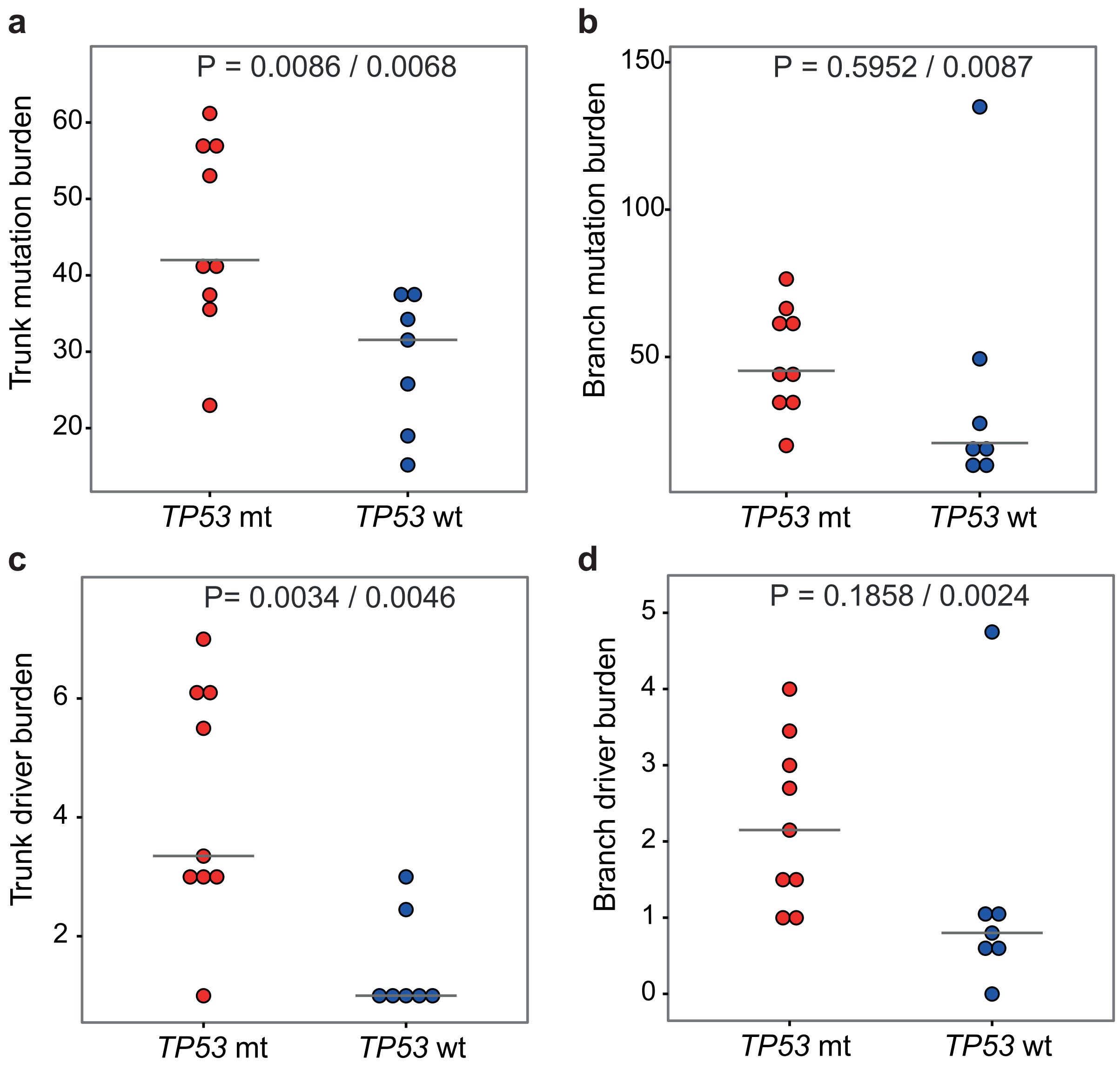

Supplementary Figure 15. *TP53*-mutant tumors have more mutations and drivers. Comparison of (**a**) trucal and (**b**) non-truncal (branch+private) mutation burdens and driver burdens (**c**, **d** respectively; extended driver list) between the *TP53*-mutant and wildtype tumors amongst our *EGFR*-mutant cohort. Three random sectors were picked 20 times iteratively and averages are represented in panels ‘a-d’. The first p-value is taking all 16 patients into consideration and the second p-value is after eliminating the outlier A102 in the analysis. P-values are calculated using Welch’s t-test. Horizontal lines represent the respective medians for each cohort.

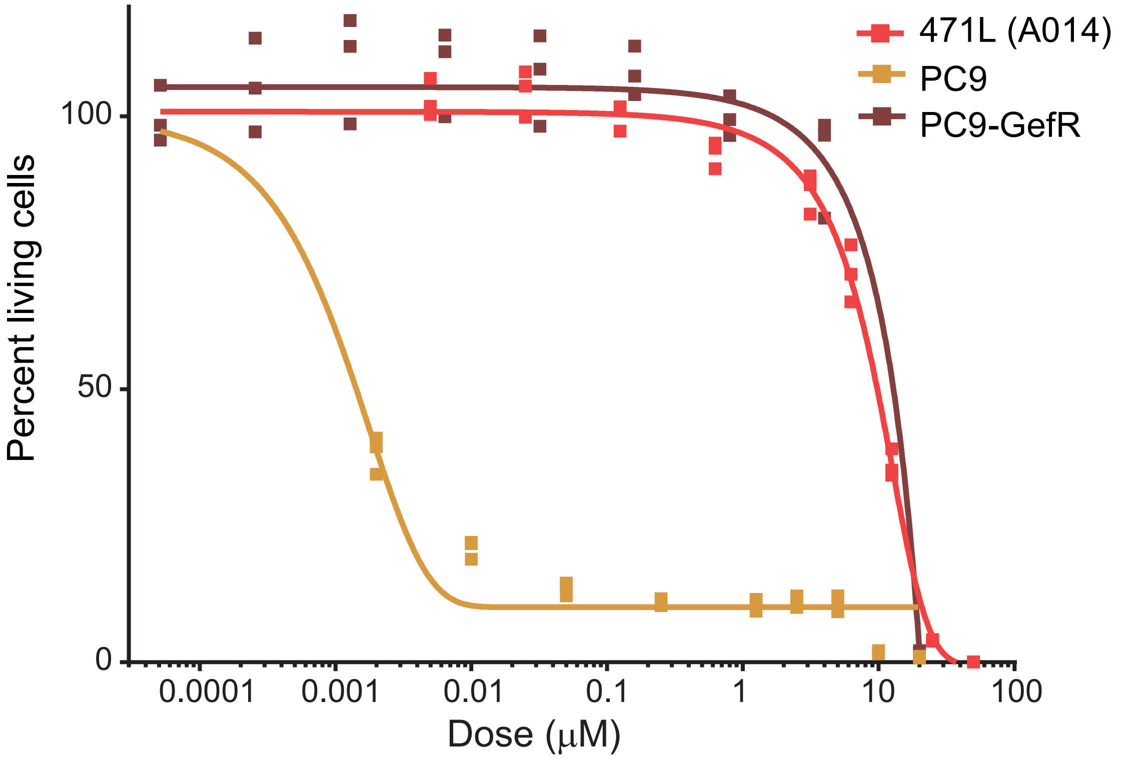

Supplementary Figure 16. A014 is a primary resistant patient. Dose response curves for Gefitinib for the A014 tumor derived cell line (471L), PC9 cell line and the isogenic Gefitinib resistant PC-9 cell line (PC9-GefR - generated inhouse).

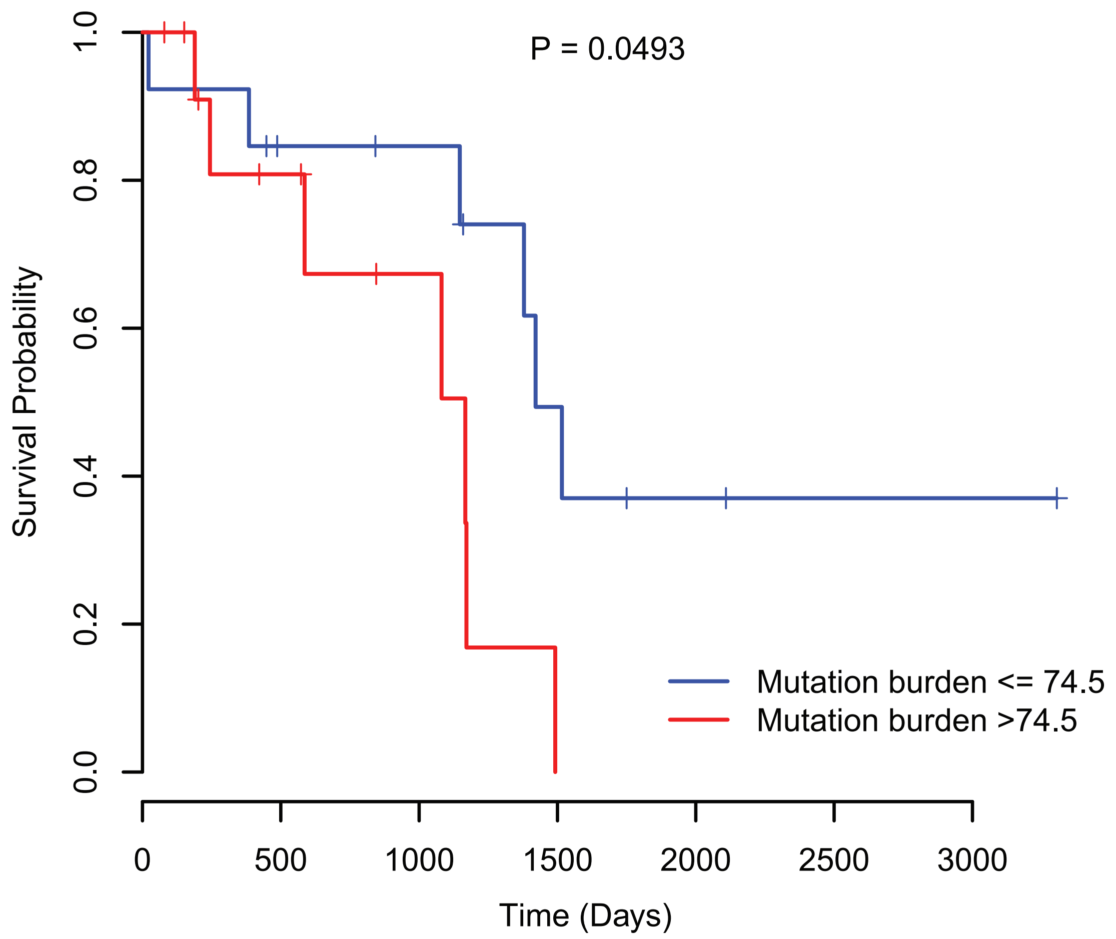

Supplementary figure 17. Mutation burden can stratify survival. Survival plots using TCGA LUAD *EGFR*-mutant cases (those with non-silent mutations in tyrosine kinase domain, n=26)[4] after stratifying above or below median number of exonic mutations (median=74.5). P-value from Chi-square test is indicated.

Supplementary Table 1: Patient information (Asian *EGFR*-mutant cohort)

Patient ID	Age	Gender	Smoking status	EGFR mutation	#Tumor sectors sequenced	Tumor type	Tumor Stage	Tumor size	Relapse status
A001	74	F	Never[#]	L858R	4	Adenocarcinoma	IB	4.0 x 3.5 x 3.0cm	
A006	57	F	Never	Exon 19 Del	6	Adenocarcinoma	IA	2.2 x 0.9 x 1.2cm	
A014	67	M	Never	L858R	11	Adenocarcinoma	IB	4.0 x 4.0 x 4.5cm	Yes
A017	56	F	Never	L858R	4	Adenocarcinoma	IIA	2.0 x 1.7 x 2.2cm	
A021	57	F	Never	L858R	5	Adenocarcinoma	IA	3.0 x 3.0 x 3.0cm	Yes
A022	64	F	Never	Exon 20 insertion	5	Adenocarcinoma	IB	4.0 x 2.7 x 2.0cm	
A027	58	F	Never	Exon 19 Del	5	Adenocarcinoma	IA	2.5 x 2.0 x 2.0cm	
A028	75	M	Never	L858R	3	Adenocarcinoma	IA	2.5 x 3.0 x 3.0cm	Yes
A062	74	M	Never	L858R	5	Adenocarcinoma	IV	4.0 x 3.0 x 2.5cm	
A065	72	F	Never	L858R	5	Adenocarcinoma	IIA	3.0 x 2.4 x 2.5 cm	Yes
A071	77	F	Never	Exon 19 Del	5	Adenocarcinoma	IA	1.5 x 1.0 x 1.5 cm	
A102	73	M	Never	L858R	4	Adenocarcinoma	IA	3.0 x 2.0 x 2.0 cm	
A103	63	F	Light ex-smoker*	L858R	4	Adenocarcinoma	IA	2.0 x 2.0 x 2.5cm	
A112	60	F	Never	Exon 20 insertion	6	Adenocarcinoma	IA	2.7 x 2.5 x 1.8 cm	
A114	61	F	Never	L858R	4	Adenocarcinoma	IIB	6.0 x 5.5 x 4.5 cm	Yes
A121	65	F	Never	Exon 19 Del	3	Adenocarcinoma	IIIA	3.0 x 2.0 x 3.0cm	

Never-smokers: less than 100 cigarettes in their lifetime

* Light ex-smokers: stopped smoking ≥ 15 years previously and smoked ≤ 10 pack-years

Supplementary Table 2: Patient information for published Caucasian dataset (only those included in analysis)[1,2]

Patient ID	Age	Gende r	Smoking status	EGFR mutation	#Tumor sectors analysed	Tumor histology	Stage
F270	62	F	Ex-smoker	-	5	Adenocarcinoma	II
F283	50	M	Ex-smoker	-	5	Adenocarcinoma	III
F292	57	F	Never	L858R	3	Adenocarcinoma	I
F324	68	F	Current smoker	-	5	Adenocarcinoma	I
F330	59	F	Ex-smoker	-	4	Adenocarcinoma	I
F339	73	M	Never	L858R	4	Adenocarcinoma	I
F356	69	F	Never	-	4	Adenocarcinoma	I
F472	74	F	Ex-smoker	-	5	Adenocarcinoma	I
F499	73	F	Ex-smoker	-	4	Adenocarcinoma	I
F4990	75	M	Ex-smoker	-	5	Adenocarcinoma	II
L001	59	F	Ex-smoker	L858R	4	Adenocarcinoma	IIA
L011	49	F	Current smoker	-	3	Adenocarcinoma	IB

Supplementary References

1. de Bruin, E.C. *et al.* Spatial and temporal diversity in genomic instability processes defines lung cancer evolution. *Science* **346**, 251-6 (2014).
2. Zhang, J. *et al.* Intratumor heterogeneity in localized lung adenocarcinomas delineated by multiregion sequencing. *Science* **346**, 256-9 (2014).
3. Cancer Genome Atlas Research, N. Comprehensive molecular characterization of clear cell renal cell carcinoma. *Nature* **499**, 43-9 (2013).
4. Cancer Genome Atlas Research, N. Comprehensive molecular profiling of lung adenocarcinoma. *Nature* **511**, 543-50 (2014).
