# Supplementary material for: Elucidating the genomic architecture of Asian EGFR-mutant lung adenocarcinoma through multi-region exome sequencing
Source: Nat Commun. 2018 Jan 15;9:216. doi: 10.1038/s41467-017-02584-z (PMC5768770; doi:10.1038/s41467-017-02584-z)
Supplement: Supplementary file 2 — Description of Additional Supplementary Files [file 41467_2017_2584_MOESM2_ESM.pdf]

Description of Supplementary Files

File Name: Supplementary Data 1
Description: List of all validated SNVs and Indels found across all samples. These are the variants used in Fig 1 and all other figures comparing mutation data across the whole manuscript.

File Name: Supplementary Data 2
Description: Patientwise list of all validated mutations to show their truncal / branch / private occurrence. This is the raw data for Supplementary Figure 2.

File Name: Supplementary Data 3
Description: This spreadsheet provides tumor purity, ploidy, whole genome doubling pvalue and TP53 mutant status of the 61 samples with SNP array data.

File Name: Supplementary Data 4
Description: This spreadsheet provides the cytoband level copy number change relative to the ploidy for the samples.

File Name: Supplementary Data 5
Description: This spreadsheet provides the gene level copy number change relative to the ploidy for the samples.

File Name: Supplementary Data 6
Description: This spreadsheet provides the cancer cell fraction and mutant allele copy number for EGFR and TP53 SNVs.

File Name: Supplementary Data 7
Description: This spreadsheet provides the GII, adGII scores for each tumor and number of trunk, branch or private driver mutations.

File Name: Supplementary Data 8
Description: This spreadsheet provides the mutation calls from targeted panel for A014 tumor sectors and the patient derived cell line.
